# Effect of dexmedetomidine on ncRNA and mRNA profiles of cerebral ischemia-reperfusion injury in transient middle cerebral artery occlusion rats model

**DOI:** 10.3389/fphar.2024.1437445

**Published:** 2024-08-07

**Authors:** Zhen Zhen Zhang, Abdul Nasir, Dong Li, Suliman Khan, Qian Bai, Feng Yuan

**Affiliations:** ^1^ Department of Anesthesiology, the Second Affiliated Hospital of Zhengzhou University, Zhengzhou, Henan, China; ^2^ Medical Research Center, the Second Affiliated Hospital of Zhengzhou University, Zhengzhou, Henan, China

**Keywords:** ischemia-reperfusion injury, dexmedetomidine, transcriptomics, non-coding RNAs, ischemic stroke

## Abstract

Ischemic stroke poses a significant global health burden, with rapid revascularization treatments being crucial but often insufficient to mitigate ischemia-reperfusion (I/R) injury. Dexmedetomidine (DEX) has shown promise in reducing cerebral I/R injury, but its potential molecular mechanism, particularly its interaction with non-coding RNAs (ncRNAs), remains unclear. This study investigates DEX’s therapeutic effect and potential molecular mechanisms in reducing cerebral I/R injury. A transient middle cerebral artery obstruction (tMACO) model was established to simulate cerebral I/R injury in adult rats. DEX was administered pre-ischemia and post-reperfusion. RNA sequencing and bioinformatic analyses were performed on the ischemic cerebral cortex to identify differentially expressed non-coding RNAs (ncRNAs) and mRNAs. The sequencing results showed 6,494 differentially expressed (DE) mRNA and 2698 DE circRNA between the sham and tMCAO (I/R) groups. Additionally, 1809 DE lncRNA, 763 DE mRNA, and 2795 DE circRNA were identified between the I/R group and tMCAO + DEX (I/R + DEX) groups. Gene ontology (GO) analysis indicated significant enrichment in multicellular biogenesis, plasma membrane components, and protein binding. KEGG analysis further highlighted the potential mechanism of DEX action in reducing cerebral I/R injury, with hub genes involved in inflammatory pathways. This study demonstrates DEX’s efficacy in reducing cerebral I/R injury and offers insights into its brain-protective effects, especially in ischemic stroke. Further research is warranted to fully understand DEX’s neuroprotective mechanisms and its clinical applications.

## 1 Introduction

Stroke remains a significant global health concern, ranking as the second leading cause of death and the third leading cause of long-term disability worldwide, with ischemic stroke constituting the majority of cases (Fan et al., 2023; Zhao Y. et al., 2023). Ischemic stroke, characterized by the obstruction of blood flow to the brain, represents approximately 70% of all stroke cases (Fan et al., 2023; Zhang R. et al., 2023). Currently, the most important treatment for acute ischemic stroke is rapid revascularization, including intravenous thrombolysis with tissue plasminogen activator (tPA) and mechanical thrombectomy, aimed at restoring blood flow to the affected brain tissue (Herpich and Rincon, 2020). Although the use of drugs and the development of thrombolytic technology can reduce nerve damage caused by ischemia, reperfusion following ischemia triggers a cascade of inflammatory reactions and oxidative stress, exacerbating tissue injury and neurological deficits, a phenomenon known as cerebral ischemia-reperfusion injury (IR) (Wu M. Y. et al., 2018). The reperfusion injury can lead to cell necrosis, apoptosis, and secondary neuron damage, further aggravating nerve function deficit (Wu M. Y. et al., 2018). Despite advancements in drug therapies and thrombolytic technologies to mitigate ischemic damage, there remain limited drugs for the effective treatment of cerebral IR injury (Paul and Candelario-Jalil, 2021), so there is still an urgent need to develop novel neuroprotective drugs to alleviate cerebral IR injury.

Dexmedetomidine (DEX), a highly selective α2 adrenergic receptor agonist, has garnered attention for its protective effect against IR injury in various organs, such as the heart, liver, lung, kidney, and brain (Zhao et al., 2020; Huang et al., 2021; Shi J. et al., 2021; Hu et al., 2022; Wang et al., 2022). In recent years, its neuroprotective properties have been increasingly recognized in diverse neurological conditions, including traumatic brain injury (TBI) (Wu J. et al., 2018; Li et al., 2020), subarachnoid hemorrhage (SAH) (Wei et al., 2022), and cerebral ischemia (Fang et al., 2021; Yin et al., 2021). The mechanism underlying the neuroprotective role of DEX involves the inhibition of inflammatory response, reducing apoptosis and autophagy, preservation of the blood-brain barrier integrity, and enhancing stable cell structure (Hu et al., 2022). Notably, DEX has been shown to modulate key signaling pathways implicated in IR, such as HIF-1α mediated autophagy inhibition (Wang et al., 2019) and NF-κB suppression (Wang et al., 2017). Furthermore, recent studies have demonstrated that DEX can reduce cerebral IR injury and play a brain-protective role by inhibiting copper inflow and iron death (Liu et al., 2022; Guo et al., 2023). However, the precise mechanism underlying the DEX neuroprotective effect in cerebral IR injury remains poorly understood and further research is still needed.

Non-coding RNAs (ncRNAs) have emerged as a pivotal regulator of gene expression and transcription regulation (Peschansky and Wahlestedt, 2014; Beermann et al., 2016). In recent years, more and more studies have shown that ncRNAs, particularly miRNAs and lncRNAs play an important role in the pathophysiology of I/R injury (Vasudeva et al., 2021; Yang et al., 2022; Xiaoqing et al., 2023). The brain is rich in ncRNAs, which regulate various central nervous system functions. Injuries such as stroke, traumatic encephalopathy, subarachnoid hemorrhage, and cerebral hemorrhage can lead to significant changes in the ncRNA expression profile. In the context of I/R injury, ncRNAs are involved in neurogenesis, angiogenesis, inflammation, and other functions, making them potential biomarkers for the diagnosis and prognosis of cerebral I/R injury (Bao et al., 2018; Wang et al., 2018; Sun et al., 2022). A variety of ncRNA, especially miRNA (such as miR-7-5p, miR-214-5p, miR-29a-3p, miR-381) and lncRNA (such as HOXA11-AS, SHNG16) have been identified as mediators in the regulation of cerebral I/R injury by DEX. These ncRNAs contribute to reducing cognitive dysfunction and improving neurodegeneration, thereby playing a neuroprotective role (Wang L. et al., 2020; Burlacu et al., 2022; Yan et al., 2023).

In this study, we aimed to investigate the impact of DEX on cerebral I/R injury using a transient middle cerebral artery occlusion (tMCAO) rats’ model. We evaluated its effects on infarct volume and neurological function and conducted whole-transcriptomic sequencing to elucidate the expression profiles of ncRNAs and mRNAs in the ischemic cortex. Bioinformatics analysis was employed to unravel the potential biological functions of differentially expressed genes and ncRNAs associated with stroke. This study provides novel insights into the pathogenesis of cerebral I/R injury and identifies potential therapeutic targets for the intervention.

## 2 Materials and methods

### 2.1 Animals

Fifty-four male Sprague-Dawley rats, aged 7–8 weeks and weighing 180–200 g, were obtained from Henan Skobes Biotechnology Co., LTD. (China). The rats were housed in standard cages with controlled environmental conditions, including a temperature of 23°C ± 1°C, humidity maintained at 60% ± 5%, and a 12-h light/dark cycle. They had *ad libitum* access to food and water and underwent a 7-day acclimatization period before experimentation. All procedures were conducted in compliance with the National Institutes of Health Guidelines for the Care and Use of Laboratory Animals and were approved by the Zhengzhou University Animal Care and Use Committee.

### 2.2 Transient middle cerebral artery occlusion (tMCAO) model establishment and experimental design

tMCAO was used to induce focal cerebral IR injury following the previously described procedure (Yuan et al., 2017a). In brief, the rats fasted for 12 h before surgery and were then anesthetized with isoflurane (4% induction, 2.5% maintenance). First, the rats were fixed to cardboard in a supine position and the neck hair was removed using a hair removal cream. Disinfect the neck with a cotton ball containing 75% ethanol. Then, a small opening of about 2 cm is made in the midline of the neck with surgical scissors, and the right common carotid artery (CCA), external carotid artery (ECA), and internal carotid artery (ICA) are separated with ophthalmic bending forceps. After proximal ligation of the CCA and ECA, the surgical wire (Beijing Cinontect Co., Ltd., Beijing, China) was inserted into the ICA until the marked black spot reached the carotid artery bifurcation at a depth of approximately 18–20 mm, indicating occlusion of the middle cerebral artery (MCA), resulting in a brief cessation of blood flow, followed by cerebral infarction in the area provided by the MCA. The skin is sutured with 5–0 silk, then after 2 h of ischemia, the filaments are extracted to initiate the reperfusion process, and the neck skin is sutured. During the whole process, the rats’ body temperature was maintained at 37°C ± 0.5°C. In the sham operation group, only the skin was cut to separate blood vessels without inserting surgical wires.

The rats were randomly assigned to three groups: Sham group (Sham), tMCAO group (I/R), and tMCAO group treated with dexmedetomidine (50 μg/kg, i. p) (I/R + DEX). DEX was dissolved in saline at the concentration of 50 μg/kg and injected intraperitoneally 30 min before MCAO. The mice in sham and tMCAO groups without drug treatment were injected with the same volume of saline. Mice were re-anesthetized and sacrificed 24 h after tMCAO. The concentration of DEX used in the experiment was based on the concentration reported in previous studies (Yuan et al., 2017b; Zhao et al., 2022; Li et al., 2023a). Each group comprised three rats for sequencing, three rats for HE staining, six rats for 2,3,5-triphenyl tetrazolium chloride (TTC) staining, and six rats for reverse transcription polymerase chain reaction (RT-PCR).

### 2.3 Behavioral testing

Neurological tests were performed 24 h post-reperfusion using a modified scoring system developed by Longa et al. (1989). Neurological function was graded on a scale of 0–5 as follows: 0 = no deficits; 1 = failure to extend left forepaw fully; 2 = circling to the left; 3 = falling to the left; 4 = no spontaneous walking with a depressed level of consciousness; and 5 = dead. The higher the score, the more severe the injury. The mice with a neurological function score of longa was 0, meaning no nerve defect, death occurred within 24 h, and subarachnoid hemorrhage found after dissection were excluded from the study.

### 2.4 Cerebral infarction analysis

The whole brain of the rat was removed immediately after anesthesia, and the brain was placed in the refrigerator at −20°C for 15 min. Then the brain was placed in the brain tank, and the brain was cut into six consecutive 2 mm coronal slices. The slices were placed in 2% TTC (2,3, 5-triphenyl tetrazolium chloride) dye solution at 37°C and incubated for 15 min, during which the slices could be turned over to make them evenly stained. After staining was completed, the sections were placed in 4% paraformaldehyde-fixed overnight and photographed. ImageJ was used to calculate the infarct area, in which the non-infarct site was dyed red, while the infarct site was not stained and turned pale by researcher who were blinded to the study group. To exclude the effect of cerebral edema, the following calculation formula was used: (contralateral hemisphere area–ipsilateral non-ischemic hemisphere area)/contralateral hemisphere area × 100% (Lu et al., 2021).

### 2.5 H&E staining

The sections were deparaffinized first, followed by washes with various concentrations of ethanol and distilled water. Following the process of xylene translucency, the segments were sealed with neutral resin and stained with H&E. Lastly, a microscope was used to observe the alterations in the brain tissue’s pathological condition. Data was gathered and the necessary sections of the sample were examined.

### 2.6 RNA-seq analysis

Total RNA was extracted from cortical cerebral samples at the infarct site using an RNA mini kit (Qiagen, Germany) following the manufacturer’s instructions and RNA quality was examined by gel electrophoresis and with Qubit (Thermo, Waltham, MA, United States). Subsequently, sequencing libraries were generated using VAHTSTM Total RNA-seq (H/M/R) Library Prep Kit for Illumina Novaseq6000. The differential gene expression analysis included transcripts and ncRNAs. Raw sequences were quality-checked with trimming (Trimmomatic 0.32) before being mapped to the Musculus genome sequence (version GRCm38.72). Gene hit counts and RPKM were calculated using CLCbio software (CLC Genomics Workbench 7.0.2, CLC Genomics Server). Using bigwig files derived from bam files, the mapped reads were visualized in the UCSC browser.

Significant Differentially expressed (DE) mRNAs and ncRNAs were defined by utilizing a cutoff of *P* < 0.05 and fold change < 1.74 [log2 (±0.8)] for subsequent analyses. DE genes (DEGs) were further processed for GO annotations and KEGG pathways significant enrichment analysis. The functional connection between the DEGs from the two regions was analyzed and elucidated using the STRING (Search Tool for the Retrieval of Interacting Genes) version: 11.0 database. The STRING database version 12 (https://string-db.org/) was utilized for the protein-protein interaction (PPI) network and hub genes were screened according to the number of neighbors. Additionally, miRNA–lncRNA–mRNA networks and miRNA–circRNA–mRNA networks were established to elucidate the intricate regulatory interactions underlying cerebral IR injury.

### 2.7 Real-time quantitative polymerase chain reaction (RT-qPCR)

The RNA sequence analysis results were verified through q-PCR. SteadyPure Quick RNA Extraction Kit (Accurate Biotechnology, Hunan, China) was used to extract total RNA, and then Evo M-MLV RT Mix Kit with gDNA Clean for qPCR (also from Accurate Biotechnology) was used to reverse-transcribe the RNA into cDNA for quantitative polymerase chain reaction. A 2 µL template was then amplified by PCR (Servicebio, Wuhan, China) using primers (Supplementary Table S1) in a reaction volume of 20 μL, including 250 nM of each primer (forward and reverse), 10 µL of SYBR Green Premix Pro Taq HS qPCR Kit (Rox Plus) (Accurate Biotechnology, Hunan, China), and 20 ng of cDNA. The reactions were performed using a 7,500 Fast Real-Time PCR Detection System (Applied Biosystems, United States) under the following conditions: 95°C for 30 s followed by 40 cycles of 95°C for 5 s and 60°C for 30 s. The ratios (I/R and I/R + DEX mRNA levels to Sham mRNA levels) were calculated using the Ct method (2^−ΔΔCT^) by normalizing all data to the housekeeping gene glyceraldehyde-3-phosphate dehydrogenase (GAPDH).

### 2.8 Statistical analysis

The data collected in this study were presented as mean ± SEM and obtained randomly. The data were statistically analyzed with a two-tailed, unpaired Student’s t-test and one-way and two-way ANOVA with repeated measures. The *post hoc* Tukey method was employed for pairwise comparisons when ANOVA showed significant differences. Statistically significant was considered when *P <* 0.05. GraphPad Prism 8.0 was used to analyze the data.

## 3 Results

### 3.1 Dexmedetomidine alleviated the neurological deficit and reduced the infarct volume in MCAO rats

**FIGURE 1:**
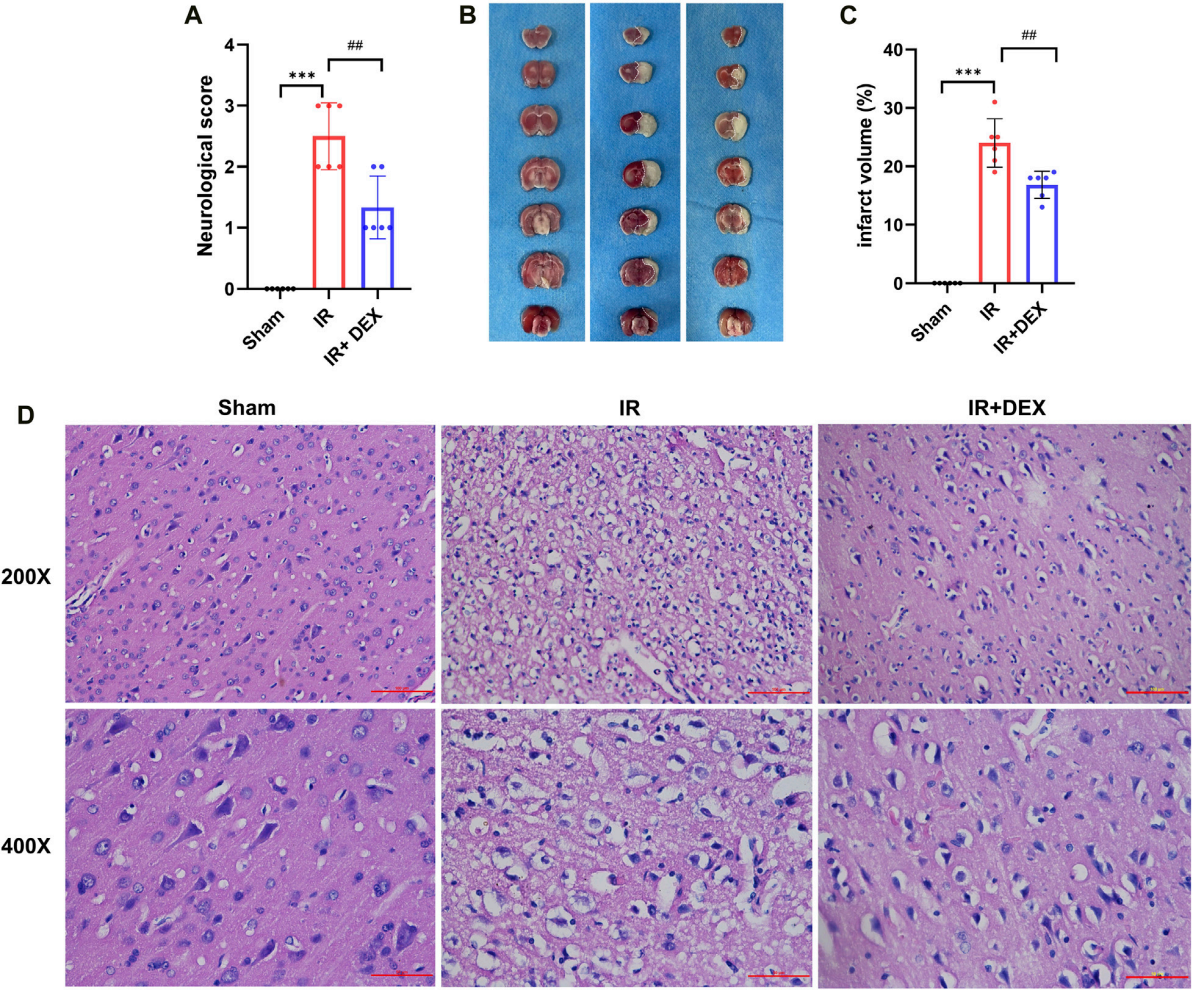
**(A)** DEX improved neurological deficit scores in tMCAO mice. ***p < 0.001 vs. sham group; ##*p* < 0.01 vs. I/R group, n = 6. **(B,C)** DEX reduced infarct volume in I/R rats. ***p < 0.001 vs. sham group; ##*p* < 0.01 vs. I/R group, n = 6. **(D)** The representative images of H&E staining in Sham, I/R, and I/R + DEX groups.

To assess the therapeutic efficacy of DEX in MCAO rats, neurological scores and TTC staining were performed 24 h after induction of cerebral ischemia and reperfusion. The neurological scores revealed a significant increase in deficits in the MCAO group compared to the sham group (*p* < 0.001). However, DEX treatment markedly improved the IR-induced neurological impairment (*p* < 0.01) when compared to the untreated MCAO group (Figure 1A).

Additionally, TTC staining was performed to assess the extent of brain damage caused by the ischemia and reperfusion. The results showed no evidence of infarction in the brain of sham rats, as demonstrated by the representative image of TTC staining (Figure 1B). In contrast, rats subjected to MCAO exhibited a substantial increase in infarct volume (24.00% ± 1.693%) compared to that in the Sham group (*p* < 0.0001) (Figure 1C). Remarkably, rats treated with DEX in conjunction with MCAO showed a significant reduction in infarct volume (16.83% ± 1.939%) compared to the untreated MCAO group (*p* < 0.005).

### 3.2 Dexmedetomidine alleviated the neuronal damage in MCAO rats

To investigate the effect of DEX on MCAO-induced rat neurons, HE staining showed no obvious pathological change in the sham brain tissue (Figure 1D). Rats subjected to MCAO exhibited the number of neuron cells decreased, the nucleolus was atrophic or disappeared, the cells showed vacuolar degeneration, inflammatory cell infiltration, and the number of denatured and necrotic neuron cells was large, suggesting severe neuron injury. Notably, in the DEX group, the neuronal damage and nerve cell necrosis decreased.

### 3.3 Expression dynamics after cerebral I/R, and DEX treatment

RNA-seq generated more than 769.3 M original reads (2.55 M in sham, 2.66 M IR group, 2.48 M IR + DEX group) on the Illumina Novaseq6000 sequencing platform. Overall, 380 M clean read lengths were obtained after removing the joint sequence and the low-quality sequence. The percentage of clean reads in each library varied from 98.41% to 99.12% in the raw data. A total of 3,251 new predictive lncRNA from the cerebral cortex were identified by screening using four analytical tools (PLEK, CPAT, CNCI, and CPC) (Supplementary Figure S1A). Our analysis further suggests that the 3,251 lncRNA were composed of 1790 (55.1%) long intergenic non-coding RNA (lncRNA), 185 (5.7%) antisense lncRNA, 1,214 (37.3%) intron lncRNA, and 62 (1.9%) bidirectional LncRNA (Supplementary Figure S1B).

Then, we analyzed DEG expression profiles in the IR group and IR + DEX group. About 7,267 and 518 significant genes were identified in 28,751 and 28,864 transcripts in the IR group and IR + DEX group, respectively, among which protein-coding RNA had the largest transcriptional changes (71.9%–72.08%), followed by known lncRNA (4.99%–5.19%). Predicted lncRNA (8.91%–9%) and other ncRNAs (13.92%–13.99%) (Supplementary Figures S1C, D). The distribution of transcript length and exons in mRNA and lncRNA is shown in Supplementary Figures S1E, F.

### 3.4 Expression of mRNA and ncRNA in the cerebral cortex after IR injury and DEX treatment

**FIGURE 2:**
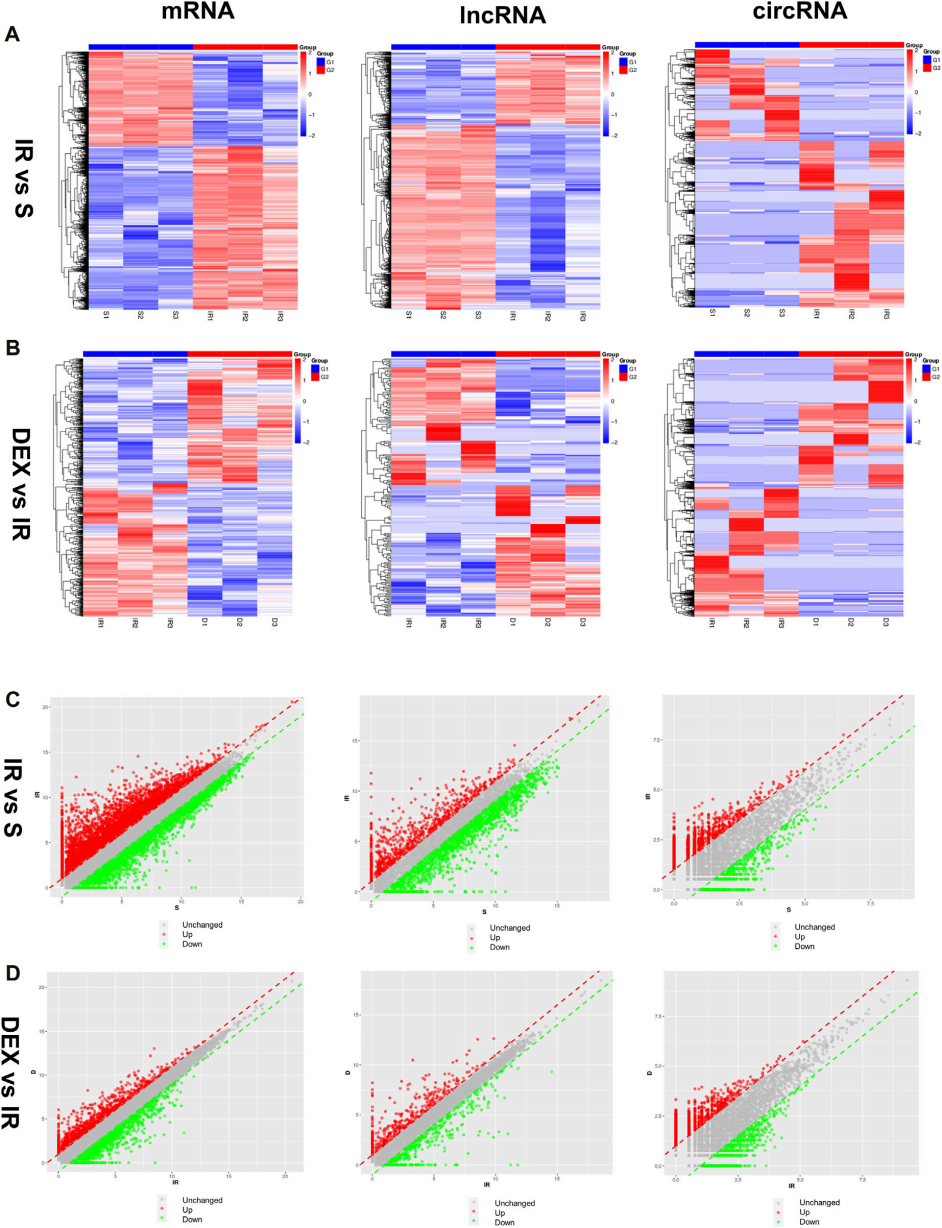
Differentially expressed mRNA, lncRNA, and circRNA in I/R and DEX after tMCAO **(A,B)** Scatter diagrams and **(C,D)** Heatmaps of significantly differentially expressed mRNA, lncRNA, and circRNA in I/R and DEX from rats after tMCAO. n = 3 rats/group.

The expression of mRNA, lncRNA, and circRNA in the cerebral infarction area of rats was significantly changed after tMACO. Approximately 6,494 mRNA (4,110 upregulated and 2,384 downregulated), 1,809 lncRNA (513 upregulated and 1,296 downregulated), and 2,698 circRNA (1740 upregulated and 958 downregulated) were significantly altered in the I/R group as compared to sham group. In the case of the I/R + DEX group, approximately 763 mRNA (369 upregulated and 394 downregulated), 232 lncRNA (118 upregulated and 114 downregulated), and 2,795 circRNA (1,395 upregulated and 1,400 downregulated) were significantly altered as compared to I/R, as shown in Figures 2A–D.

### 3.5 Functional enrichment analysis of differential genes

We conducted Gene Ontology (GO) analysis to explore the functional characteristics of differential expressed mRNA in the I/R and IR + DEX groups. The analysis revealed significant enrichment in biological processes (BPs), including the regulation of multicellular organismal processes, the biological development process of cells and systems, the immune regulation process, and the regulation of nociceptive stimuli. Notably, we observed Cellular components (CC) are enriched in cellular and neuronal projection, and the most robust molecular functions (MFs) are enriched in protein and receptor binding. Similarly, circRNA colocalization and co-expression genes analysis demonstrated analogous enrichment, while trans-lncRNA analysis highlighted involvement in cell and tissue genesis and regulation, particularly in neuronal projection, cell junction, cell projection, synapses, axons and dendrites (Figures 5A, B).

**FIGURE 3:**
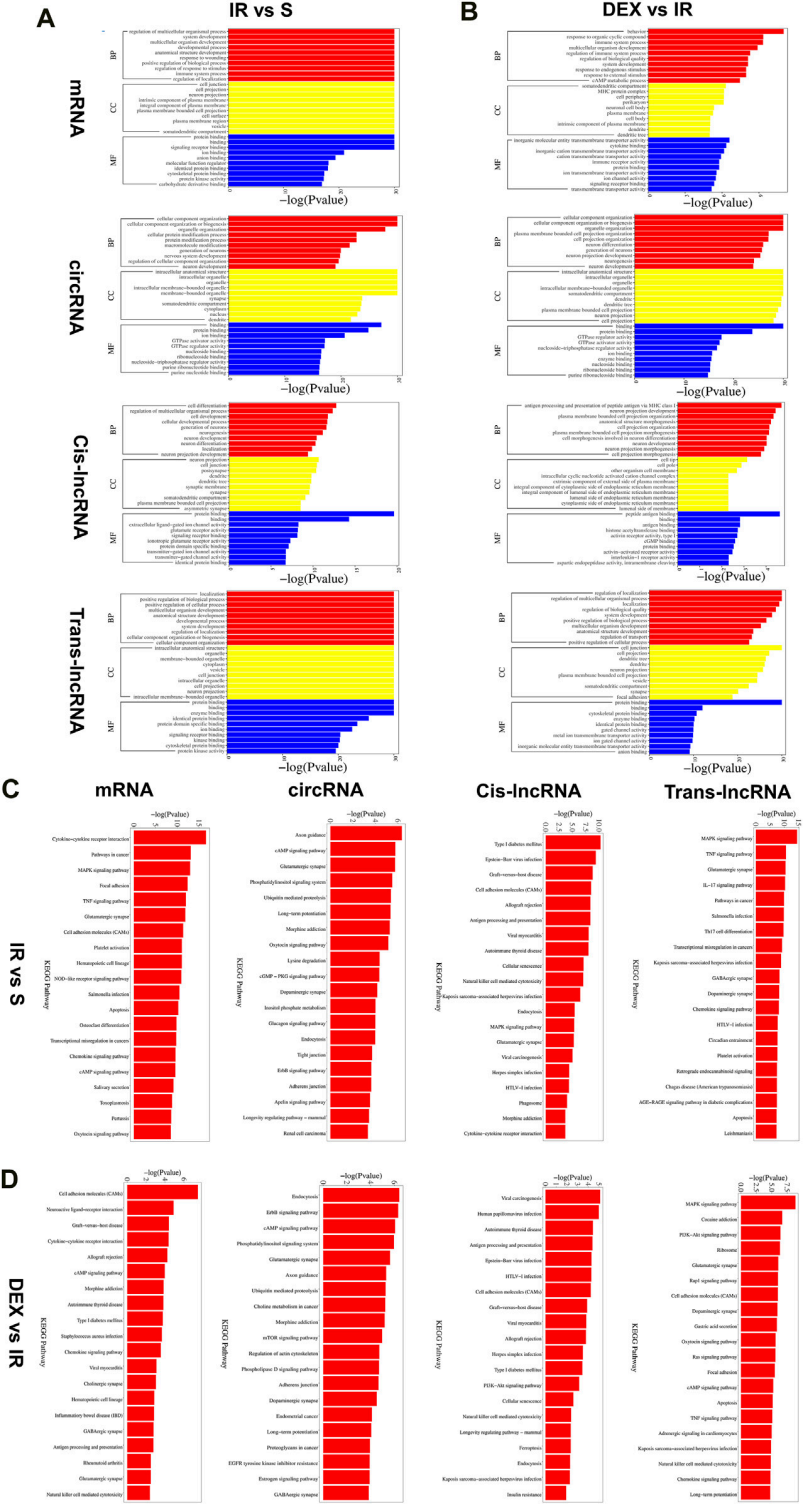
Functional prediction of DE mRNAs, DE circRNAs, DE cis-lncRNAs, DE trans-lncRNAs in I/R **(A)** and I/R + DEX **(B)**, by GO analyses. Analysis of the Gene Ontology database graphically displays the top 10 significant GO enrichment results with the candidate targeted genes in the biological process, molecular function, and cellular component in the I/R and I/R + DEX. Functional prediction of DE mRNAs, DE circRNAs, DE cis-lncRNAs, and DE trans-lncRNAs in I/R **(C)** and I/R + DEX **(D)**, by KEGG analyses showed top 20 enrichments of KEGG pathways. The abscissa represents -log10 (*p*-value). The more significant the abscissa is, the more significant the pathway enrichment (*p* ≤ 0.05).

Furthermore, KEGG pathway analysis of DE RNA showed significant enrichment in pathways such as cytokine−cytokine receptor interaction, glutamatergic, synapse, cell adhesion molecules (CAMs), hematopoietic, cell lineage, chemokine signaling pathway and cAMP signaling pathway. DE circRNA analysis displayed enrichment in axon guidance, cAMP signaling pathway, glutamatergic synapse, phosphatidylinositol signaling system, ubiquitin-mediated proteolysis, long-term potentiation, morphine addiction, dopaminergic synapse, and endocytosis. Additionally, DE cis-lncRNA pathways included type I diabetes mellitus, epstein−Barr virus infection, graft−versus−host disease, and antigen processing and presentation, while DE trans-lncRNA pathways involved dopaminergic synapse, apoptosis, TNF signaling pathway and chemokine signaling pathway (Figures 3C, D).

### 3.6 Competing endogenous RNA (CeRNA) network analysis

**FIGURE 4:**
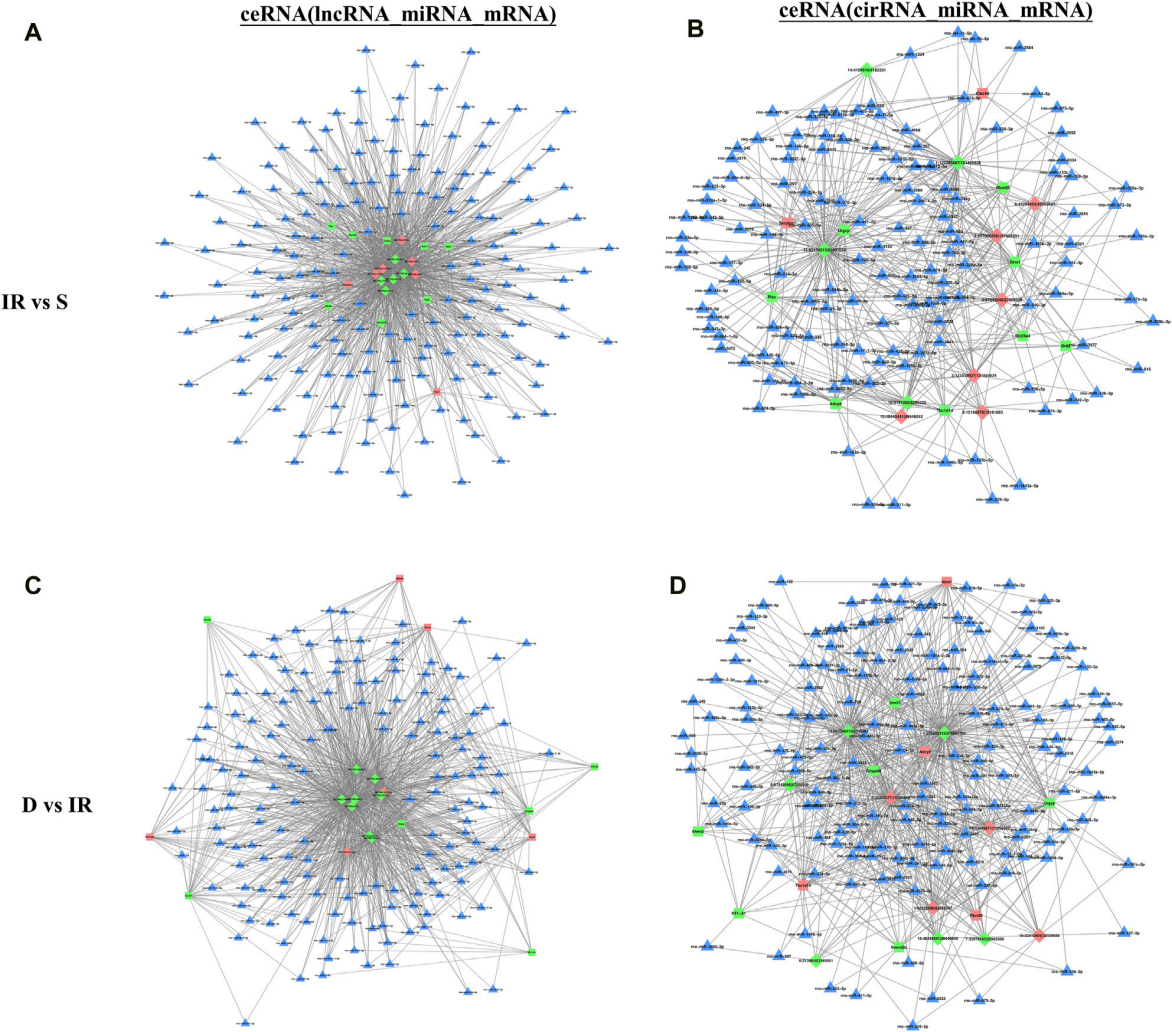
The top 10 lncRNAs and circRNAs and their target genes are displayed according to their respective network degrees of mRNA, lncRNA, and circRNAs. **(A,C)** ceRNA (lncRNA–miRNA–mRNA) network on I/R **(A)** and I/R + DEX **(C)**. **(B,D)** ceRNA (circRNA–miRNA–mRNA) network on I/R **(B)** and I/R + DEX **(D)**. Red represents upregulation, green represents downregulation, and blue represents unknown. Triangle: miRNA; Square: mRNA; Diamond: lncRNA or circRNA.

To identify the interaction between DE RNA in the I/R and the I/R + DEX group and predict their regulatory relationship, we constructed the ceRNA network diagram comprising the top 10 lncRNA-miRNA-mRNA and circRNA-miRNA-mRNA interactions based on correlation analysis. Through node analysis, we identified MSTRG.17966.6, MSTRG.288.3, MSTRG.288.2, MSTRG.2024.4, and specific loci like 9:67242046|67269326 (rno-Abi2_0020), and 15:48443441|48446043 (rno-intergenic_000444), which exhibited simultaneous interaction in both I/R and I/R + DEX groups with multiple miRNA and mRNA. These findings suggest the potential significance of these lncRNA/circRNA in the DEX treatment of cerebral I/R injury. Moreover, the establishment of co-expression networks serves as a foundation for further research exploration into the functional roles of ceRNAs and the lncRNA/circRNA-miRNA-mRNA axis in the pathophysiology of cerebral I/R injury. Notably, rno-miR-24-3p and rno-miR-6333 were found as key binding targets in the constructed lncRNA/circRNA-miRNA-mRNA axis. For instance, the MSTRG.288.3/rno-miR-6333/*Grm1* axis, MSTRG.17966.6/rno-miR-24-3p/*URGCP* axis, and rno-Abi2_0020/rno-miR-24-3p/*Plec* axis are potential regulatory axes modulated by DEX to reduce cerebral I/R injury (Figure 4).

### 3.7 DEX reduces cerebral I/R injury by reducing inflammation and oxidative stress

**FIGURE 5:**
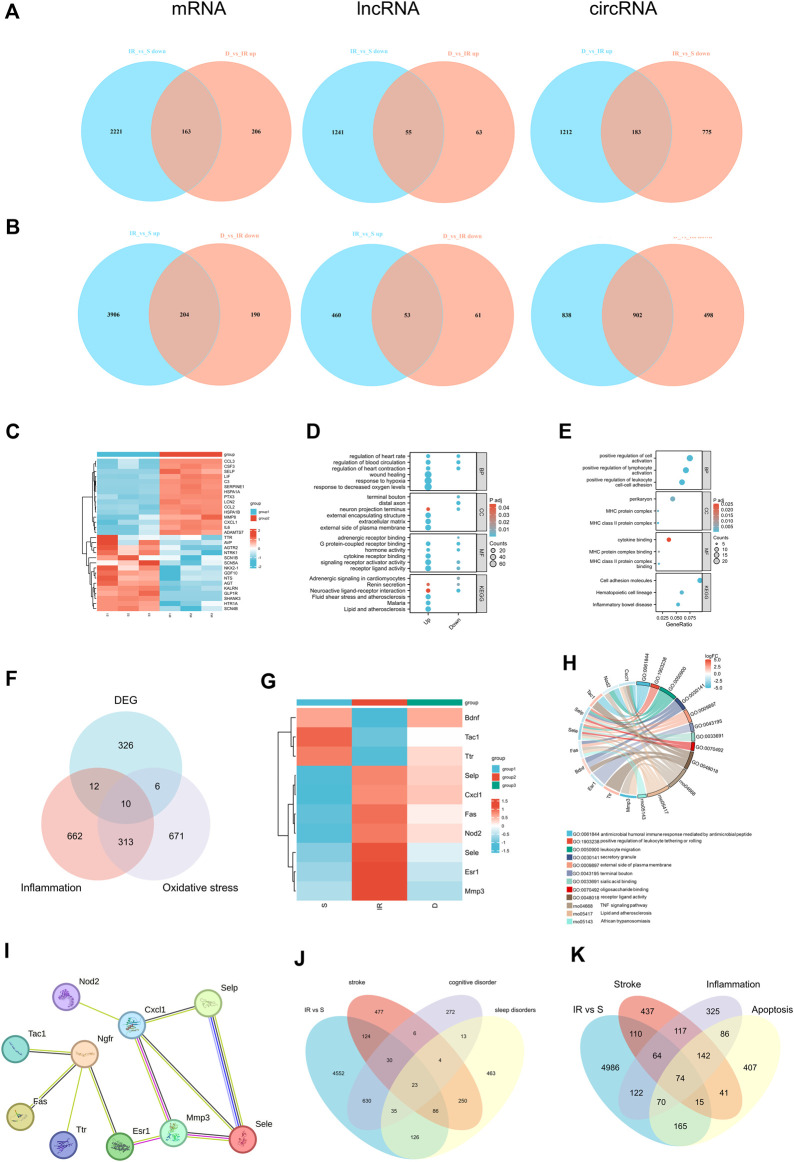
**(A)** Venn diagrams indicate the downregulated mRNA, lncRNA, circRNA in I/R and upregulated in I/R + DEX, **(B)**Venn diagrams indicate the upregulated mRNA, lncRNA, circRNA in I/R and downregulated in I/R + DEX. n = 3 rats/group. **(C)** Heat map of the top 15 up- and downregulated genes of stroke in the I/R group, **(D)** and their GO and KEGG functional enrichment, **(E)** GO and KEGG functional enrichment analysis of reverse effect genes after DEX application, **(F)** Venn diagrams **(G)** Heat map **(H)** Functional enrichment analysis, and **(I)** PPI network analysis indicate the number of genes associated with both inflammation and oxidative stress. **(J)** Venn diagrams indicate the number of DEGs mapped to stroke, cognitive disorder, and sleep disorders and **(K)** the number of DEGs mapped to stroke, inflammation- and apoptosis-related genes in the I/R group.

We employed Venn diagrams to show the genes whose expression was reversed after DEX injection. In the IR + DEX group, compared to IR alone, 204 mRNA, 53 lncRNA, and 902 circRNA were downregulated, while 163 mRNA, 55 lncRNA, and 183 circRNA were upregulated, suggesting their relevance to DEX-mediated neuroprotection (Figures 5A, B). Notably, analysis of 341 DE mRNA associated with stroke (259 upregulated, 82 downregulated) in the IR group (Supplementary Figure S2) revealed upregulation of genes like *Ccl2, Cxcl1, C3, Serpine1, Adamts7, Csf3, Ptx3, MMP8, Lif*, and *Lcn2*, and downregulation of *Gdf10*, *Agtr2* and *Shank3* (Figure 5C). GO and KEGG enrichment analysis underscored hypoxia, regulation of blood circulation, and wound healing among these genes. Subsequent analysis of 367 DE mRNA reversed by DEX treatment showed significant enrichment in inflammatory pathways such as leukocyte adhesion and immune complex formation (Figures 5D, E). Neuroinflammation and oxidative stress play an important role in I/R injury, and DEX has anti-inflammatory and anti-oxidative stress effects. Therefore, we used gene cards to characterize the above 367 DE mRNA. Gene characterization revealed associated with neuroinflammation (22 DE mRNA) and oxidative stress (16 DE mRNA). Among these, 10 DE mRNA are associated with both neuroinflammation and oxidative stress: *Selp*, *Cxcl1, Fas, Sele, Esr1, Nod2, Mmp3, Tac1, Ttr,* and *Bdnf* (Figures 5F, G). These genes participate in common biological processes such as leukocyte migration and response to external stimuli while exhibiting enrichment in cell components like perikaryon and the external side of the plasma membrane. Additionally, their molecular functions are enriched in signal receptors and chemokine binding. KEGG analysis identified the TNF signaling pathway and cytokine interaction as significantly enriched pathways, underscoring their role in cerebral I/R injury (Figure 5H). The STRING database was used for 10 significant differentially expressed mRNAs, which are identified as associated with both neuroinflammation and oxidative stress to establish the PPI network as shown in Figure 5I. *Cxcl1* showed co-expression with *Selp*, *Sele*, and *Mmp3*, with co-expression scores were 0.657, 0.629, and 0.728, respectively. *Ngfr* showed co-expression with *Tac1*, *Fas*, and Esr, and co-expression scores were 0.533, 0.47, and 0.556 respectively. These findings implicate DEX in mitigating neuroinflammation and oxidative stress, offering promising therapeutic avenues for cerebral I/R injury.

### 3.8 DEGs have shown cognitive dysfunction and sleep disorders are associated with cerebral I/R injury

DEGs associated with stroke, cognitive dysfunction, and sleep disorders after I/R injury were characterized using Gene Cards databases. Analysis of 6494 DE mRNA in the I/R group showed relevance to stroke, cognitive dysfunction, and sleep disorder, with 53 DEGs associated with stroke and cognitive dysfunction, 109 with sleep disorders, and 23 with all three conditions (Figure 5J). Further characterization via Venn diagram showed overlapping DEGs involved in neuroinflammation, apoptosis, and stroke. Specifically, 74 DEGs were associated with neuroinflammation, apoptosis, and stroke, while 138 DEGs were linked to stroke and neuroinflammation. Additionally, 89 DEGs were related to stroke and apoptosis, and 74 genes were associated with stroke, neuroinflammation, and apoptosis (Figure 5K). The proportion of these differential genes in stroke, neuroinflammation, apoptosis, cognitive dysfunction, and sleep disorders in the I/R group was 3.50%, 4.39%, 4.31%, 9.56%, and 3.59%, respectively (Supplementary Figure S2).

### 3.9 Validation of genes reversed by DEX

**FIGURE 6:**
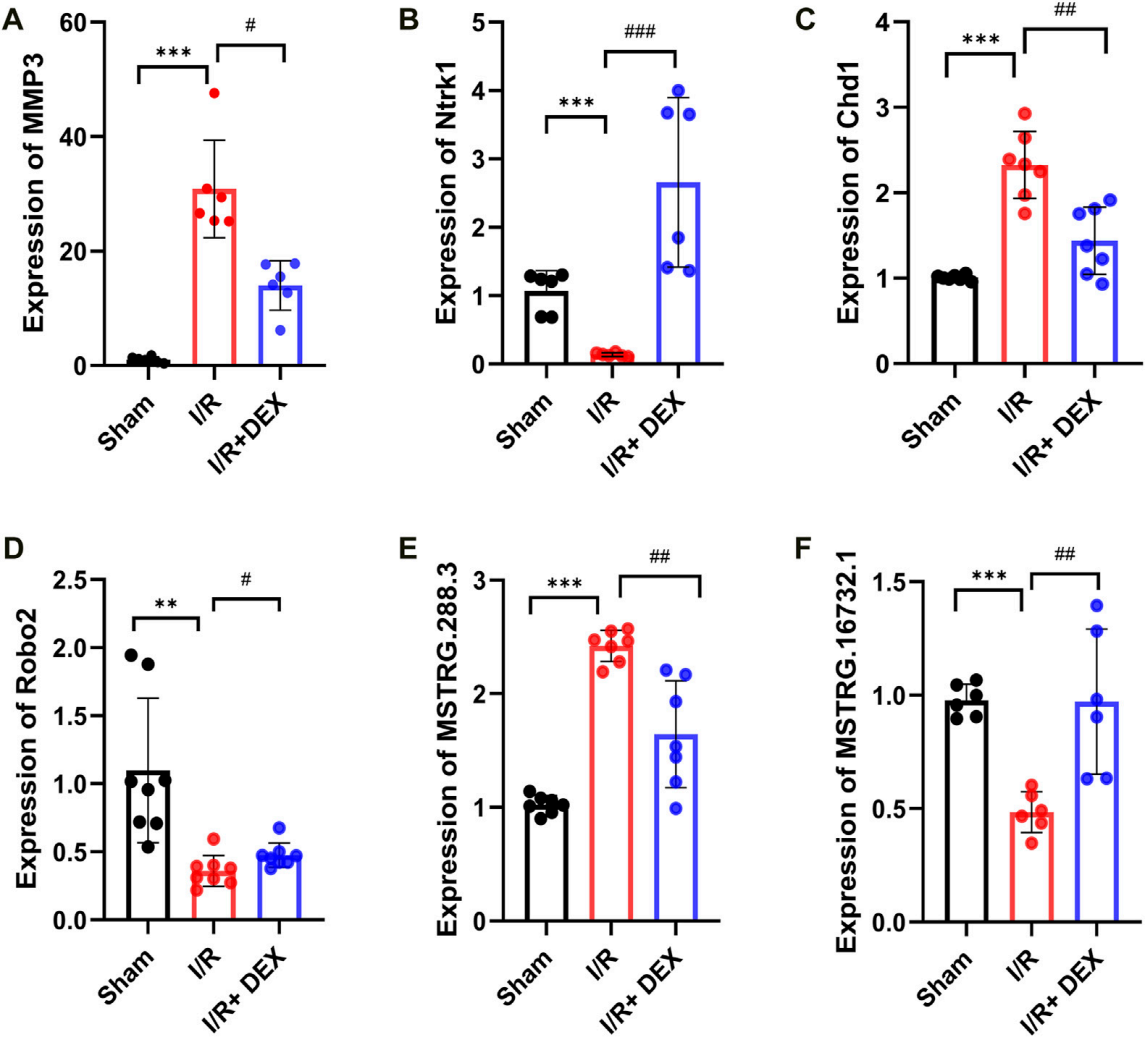
Validations of DE mRNA, circRNAs, and lncRNAs, in I/R and I/R + DEX after tMCAO. The expression levels of mRNAs MMP3 and Ntrk1 **(A,B)**, circRNA Chd1 and Robo2 **(C,D)**, lncRNA MSTRG.288.3 and MSTRG.16732.1 **(E,F)**. */#*p* < 0.05, **/##*p* < 0.01, ***/###*p* < 0.001, n = 6.

To verify the reliability of the sequencing results, we selected two mRNA (MMP3, Ntrk1), two circRNAs (Chd1, Robo2), and two lncRNAs (MSTRG.288.3, MSTRG.16732.1), we found that the results were consistent with sequencing results (Figures 6A–F).

## 4 Discussion

In this study, we established a clinically relevant model of I/R injury in rats and found that DEX significantly improved neurological impairment and reduced infarct volume. Non-treated rats exhibited increased infarct volumes and neurological deficits. Consistent with previous studies (Yuan et al., 2017a; Wang et al., 2019; Zhai et al., 2019), DEX reduced cerebral infarction size, improved motor dysfunction, and decreased neuronal damage. Notably, this study is the first to use full transcriptome sequencing and bioinformatic analysis to observe the expression profile of ncRNA in the cortex of infarct sites in rats after DEX treatment.

To predict gene functions of DE mRNA and ncRNA in I/R injury, we performed GO and KEGG enrichment analyses of these DEGs. The results highlighted significant roles in neuronal production and differentiation, response to stimulation, immune response, MAPK signaling pathway, cytokine-to-cytokine binding, cAMP signaling pathway, and TNF signaling pathway. These findings are consistent with previous reports (Dergunova et al., 2018). Many DEGs were associated with neuroinflammation and oxidative stress, key contributors to cerebral I/R injury (Wu et al., 2020). We constructed ceRNA networks for the top 10 DEGs, predicting targeted regulatory relationships and identifying potential therapeutic targets for I/R.

Increased oxygen supply after I/R leads to oxidative stress and increased release of inflammatory factors, triggering a cascade of reactions resulting in apoptosis, disruption of the blood-brain barrier (BBB), cerebral edema, and bleeding transformation (Jurcau and Simion, 2021). Anti-inflammatory as a potential therapeutic target for I/R injury has been studied for a long time (Tirandi et al., 2023). Various cytokines and signaling pathways, including NF-κB, MAPK, and PI3K-AKT, are implicated in cerebral I/R injury. Our study identified stroke-related molecules such as *Ccl2* (Dimitrijevic et al., 2006; Stowe et al., 2012; Geng et al., 2022; Georgakis et al., 2022), *Cxcl1* (Shi Y. et al., 2021), *C3* (Mocco et al., 2006; Yang et al., 2013; Yang et al., 2021; Hou et al., 2022), *Serpine1* (Pu et al., 2022; Palakurti et al., 2023), *Adamts7* (Sharifi et al., 2023), *Csf3* (Garcia-Bonilla et al., 2015), *Ptx3* (Rodriguez-Grande et al., 2014; Rodriguez-Grande et al., 2015; Shindo et al., 2016; de Oliveira et al., 2019; Shindo et al., 2021), *Mmp8* (Han et al., 2016; Ren et al., 2020), *Lif* (Cai et al., 2022), and *Lcn2* (Jin et al., 2014; Zhao et al., 2019; Wang G. et al., 2020; Li et al., 2023b; Zhao R. Y. et al., 2023) were significantly upregulated after I/R, whereas *Gdf10* (Li et al., 2015), *Agtr2* (Iwai et al., 2004; Iwanami et al., 2011), *Shank3* (Datta et al., 2011; Zhang et al., 2024) downregulated after I/R, aligning with existing literature.

DEX, a highly selective α2 adrenergic receptor agonist, has notable anti-inflammatory and anti-oxidative properties. By reducing cerebral blood flow and metabolic oxygen demand, DEX slightly lowers intracranial pressure. Its neuroprotective effects have been demonstrated in multiple brain injury models, primarily by reducing cerebral catecholamine release. Our high-throughput sequencing identified 367 genes whose expression was reversed by DEX treatment. GO and KEGG analyses identified these genes are involved in inflammatory and immune biological processes. We identified DEGs associated with both inflammation and oxidative stress, including *Selp, Cxcl1, ESR1, NOD2, Fas*, and *BDNF*, suggesting they play crucial roles in DEX’s neuroprotective mechanism. For instance, selectin (*Selp, Sele*), cell adhesion molecules, are upregulated following pro-inflammatory stimuli (Cappenberg et al., 2022) and promote thrombosis (Purdy et al., 2022). DEX inhibited the I/R-induced increase in *Selp* and *Sele* expression, potentially contributing to its protective effects. *Cxcl1*, neutrophil chemokine, is involved in post-ischemic inflammation and pain (Huang et al., 2023). DEX can reverse *Cxcl* increase, mitigating inflammation in various models (Shen et al., 2020; Zhang S. et al., 2023). *ESR1*, an estrogen receptor, mediates neuroprotection by reducing inflammation (Suzuki et al., 2009). DEX reversed the I/R-induced upregulation of *ESR1,* though its precise regulatory mechanism needs further research. *NOD2*, involved in I/R inflammatory response, exacerbates injury when activated (Liu et al., 2015). DEX’s modulation of *NOD2*’s role in I/R injury remains to be elucidated. *Fas*, an apoptotic receptor, increases in ischemic stroke and contributes to neuronal apoptosis (Chojnowski et al., 2023). DEX’s reduction of *Fas* expression supports its anti-apoptotic effects. Lastly, *BDNF* promotes neuronal survival and plasticity. DEX enhanced *BDNF* levels, supporting neuroprotection (Li et al., 2018; Chen et al., 2024).

Our study still had some limitations, first transcriptome analysis of differential genes was limited to a single time point, necessitating multi-time-point analyses. Second, our RNA-seq mainly tests the expression profiles of mRNA, lncRNA, and circRNA, but no miRNA. Further research is needed to validate whether these DEGs can serve as therapeutic targets.

## 5 Conclusion

To summarize, this study demonstrated that DEX significantly reduces I/R injury in a rat model by improving neurological outcomes and reducing infarct volumes. Through comprehensive transcriptome sequencing and bioinformatic analysis, we identified significant changes in the expression profiles of mRNAs and ncRNAs in the cortex of infarct sites. The study provides new insights into the molecular mechanisms of DEX’s protective effects against cerebral I/R injury, identifying several potential therapeutic targets. However, further research is needed to validate these findings and explore the therapeutic potential of these DEGs. These findings provide a basis for exploring DEX’s neuroprotective mechanisms and developing new therapies for I/R injury.

## Data Availability

The data presented in the study are deposited in the https://www.ncbi.nlm.nih.gov/, accession GSE268634.

## References

[B1] Bao M. H. Szeto V. Yang B. B. Zhu S. Z. Sun H. S. Feng Z. P. (2018). Long non-coding RNAs in ischemic stroke. Cell. Death Dis. 9 (3), 281. 10.1038/s41419-018-0282-x 29449542 PMC5833768

[B2] Beermann J. Piccoli M. T. Viereck J. Thum T. (2016). Non-coding RNAs in development and disease: background, mechanisms, and therapeutic approaches. Physiol. Rev. 96 (4), 1297–1325. 10.1152/physrev.00041.2015 27535639

[B3] Burlacu C. C. Neag M. A. Mitre A. O. Sirbu A. C. Badulescu A. V. Buzoianu A. D. (2022). The role of miRNAs in dexmedetomidine’s neuroprotective effects against brain disorders. Int. J. Mol. Sci. 23 (10), 5452. 10.3390/ijms23105452 35628263 PMC9141783

[B4] Cai W. Shi L. Zhao J. Xu F. Dufort C. Ye Q. (2022). Neuroprotection against ischemic stroke requires a specific class of early responder T cells in mice. J. Clin. Investig. 132 (15), e157678. 10.1172/JCI157678 35912857 PMC9337834

[B5] Cappenberg A. Kardell M. Zarbock A. (2022). Selectin-mediated signaling-shedding light on the regulation of integrin activity in neutrophils. Cells 11 (8), 1310. 10.3390/cells11081310 35455989 PMC9025114

[B6] Chen X. Chen A. Wei J. Huang Y. Deng J. Chen P. (2024). Dexmedetomidine alleviates cognitive impairment by promoting hippocampal neurogenesis via BDNF/TrkB/CREB signaling pathway in hypoxic-ischemic neonatal rats. CNS Neurosci. Ther. 30 (1), e14486. 10.1111/cns.14486 37830170 PMC10805444

[B7] Chojnowski K. Opiełka M. Gozdalski J. Radziwon J. Dańczyszyn A. Aitken A. V. (2023). The role of arginine-vasopressin in stroke and the potential use of arginine-vasopressin type 1 receptor antagonists in stroke therapy: a narrative review. Int. J. Mol. Sci. 24 (3), 2119. 10.3390/ijms24032119 36768443 PMC9916514

[B8] Datta A. Jingru Q. Khor T. H. Heese K. Sze S. K. (2011). Quantitative neuroproteomics of an *in vivo* rodent model of focal cerebral ischemia/reperfusion injury reveals a temporal regulation of novel pathophysiological molecular markers. J. Proteome Res. 10 (11), 5199–5213. 10.1021/pr200673y 21950801

[B9] de Oliveira T. H. C. Souza D. G. Teixeira M. M. Amaral F. A. (2019). Tissue dependent role of PTX3 during ischemia-reperfusion injury. Front. Immunol. 10, 1461. 10.3389/fimmu.2019.01461 31354697 PMC6635462

[B10] Dergunova L. V. Filippenkov I. B. Stavchansky V. V. Denisova A. E. Yuzhakov V. V. Mozerov S. A. (2018). Genome-wide transcriptome analysis using RNA-Seq reveals a large number of differentially expressed genes in a transient MCAO rat model. BMC Genomics 19 (1), 655. 10.1186/s12864-018-5039-5 30185153 PMC6125876

[B11] Dimitrijevic O. B. Stamatovic S. M. Keep R. F. Andjelkovic A. V. (2006). Effects of the chemokine CCL2 on blood-brain barrier permeability during ischemia-reperfusion injury. J. Cereb. Blood Flow. Metab. 26 (6), 797–810. 10.1038/sj.jcbfm.9600229 16192992

[B12] Fan J. Li X. Yu X. Liu Z. Jiang Y. Fang Y. (2023). Global burden, risk factor analysis, and prediction study of ischemic stroke, 1990-2030. Neurology 101 (2), e137–e150. 10.1212/WNL.0000000000207387 37197995 PMC10351546

[B13] Fang H. Li H. F. Yan J. Y. Yang M. Zhang J. P. (2021). Dexmedetomidine-up-regulated microRNA-381 exerts anti-inflammatory effects in rats with cerebral ischaemic injury via the transcriptional factor IRF4. J. Cell. Mol. Med. 25 (4), 2098–2109. 10.1111/jcmm.16153 33314611 PMC7882963

[B14] Garcia-Bonilla L. Racchumi G. Murphy M. Anrather J. Iadecola C. (2015). Endothelial CD36 contributes to postischemic brain injury by promoting neutrophil activation via CSF3. J. Neurosci. 35 (44), 14783–14793. 10.1523/JNEUROSCI.2980-15.2015 26538649 PMC4635129

[B15] Geng H. Chen L. Tang J. Chen Y. Wang L. (2022). The role of CCL2/CCR2 Axis in cerebral ischemia-reperfusion injury and treatment: from animal experiments to clinical trials. Int. J. Mol. Sci. 23 (7), 3485. 10.3390/ijms23073485 35408846 PMC8998625

[B16] Georgakis M. K. Bernhagen J. Heitman L. H. Weber C. Dichgans M. (2022). Targeting the CCL2-CCR2 axis for atheroprotection. Eur. Heart J. 43 (19), 1799–1808. 10.1093/eurheartj/ehac094 35567558

[B17] Guo Q. Ma M. Yu H. Han Y. Zhang D. (2023). Dexmedetomidine enables copper homeostasis in cerebral ischemia/reperfusion via ferredoxin 1. Ann. Med. 55 (1), 2209735. 10.1080/07853890.2023.2209735 37162502 PMC10173798

[B18] Han J. E. Lee E. J. Moon E. Ryu J. H. Choi J. W. Kim H. S. (2016). Matrix metalloproteinase-8 is a novel pathogenetic factor in focal cerebral ischemia. Mol. Neurobiol. 53 (1), 231–239. 10.1007/s12035-014-8996-y 25421209

[B19] Herpich F. Rincon F. (2020). Management of acute ischemic stroke. Crit. Care Med. 48 (11), 1654–1663. 10.1097/CCM.0000000000004597 32947473 PMC7540624

[B20] Hou J. Y. Cao G. Z. Tian L. L. Zhou R. Zhang Y. Xu H. (2022). Integrated transcriptomics and metabolomics analysis reveals that C3 and C5 are vital targets of DuZhi Wan in protecting against cerebral ischemic injury. Biomed. Pharmacother. 155, 113703. 10.1016/j.biopha.2022.113703 36126455

[B21] Hu Y. Zhou H. Zhang H. Sui Y. Zhang Z. Zou Y. (2022). The neuroprotective effect of dexmedetomidine and its mechanism. Front. Pharmacol. 13, 965661. 10.3389/fphar.2022.965661 36204225 PMC9531148

[B22] Huang X. Guo M. Zhang Y. Xie J. Huang R. Zuo Z. (2023). Microglial IL-1RA ameliorates brain injury after ischemic stroke by inhibiting astrocytic CXCL1-mediated neutrophil recruitment and microvessel occlusion. Glia 71 (7), 1607–1625. 10.1002/glia.24359 36929654

[B23] Huang Y. Q. Wen R. T. Li X. T. Zhang J. Yu Z. Y. Feng Y. F. (2021). The protective effect of dexmedetomidine against ischemia-reperfusion injury after hepatectomy: a meta-analysis of randomized controlled trials. Front. Pharmacol. 12, 747911. 10.3389/fphar.2021.747911 34712138 PMC8546301

[B24] Iwai M. Liu H. W. Chen R. Ide A. Okamoto S. Hata R. (2004). Possible inhibition of focal cerebral ischemia by angiotensin II type 2 receptor stimulation. Circulation 110 (7), 843–848. 10.1161/01.CIR.0000138848.58269.80 15289370

[B25] Iwanami J. Mogi M. Tsukuda K. Min L. J. Sakata A. Jing F. (2011). Effect of angiotensin II type 2 receptor deletion in hematopoietic cells on brain ischemia-reperfusion injury. Hypertension 58 (3), 404–409. 10.1161/HYPERTENSIONAHA.111.177873 21768524

[B26] Jin M. Kim J. H. Jang E. Lee Y. M. Soo Han H. Woo D. K. (2014). Lipocalin-2 deficiency attenuates neuroinflammation and brain injury after transient middle cerebral artery occlusion in mice. J. Cereb. Blood Flow. Metab. 34 (8), 1306–1314. 10.1038/jcbfm.2014.83 24780901 PMC4126090

[B27] Jurcau A. Simion A. (2021). Neuroinflammation in cerebral ischemia and ischemia/reperfusion injuries: from pathophysiology to therapeutic strategies. Int. J. Mol. Sci. 23 (1), 14. 10.3390/ijms23010014 35008440 PMC8744548

[B28] Li H. Lu C. Yao W. Xu L. Zhou J. Zheng B. (2020). Dexmedetomidine inhibits inflammatory response and autophagy through the circLrp1b/miR-27a-3p/Dram2 pathway in a rat model of traumatic brain injury. Aging (Albany NY) 12 (21), 21687–21705. 10.18632/aging.103975 33147167 PMC7695368

[B29] Li J. Wang K. Liu M. He J. Zhang H. Liu H. (2023a). Dexmedetomidine alleviates cerebral ischemia-reperfusion injury via inhibiting autophagy through PI3K/Akt/mTOR pathway. J. Mol. Histology 54 (3), 173–181. 10.1007/s10735-023-10120-1 37186301

[B30] Li J. Xu P. Hong Y. Xie Y. Peng M. Sun R. (2023b). Lipocalin-2-mediated astrocyte pyroptosis promotes neuroinflammatory injury via NLRP3 inflammasome activation in cerebral ischemia/reperfusion injury. J. Neuroinflammation 20 (1), 148. 10.1186/s12974-023-02819-5 37353794 PMC10288712

[B31] Li S. Nie E. H. Yin Y. Benowitz L. I. Tung S. Vinters H. V. (2015). GDF10 is a signal for axonal sprouting and functional recovery after stroke. Nat. Neurosci. 18 (12), 1737–1745. 10.1038/nn.4146 26502261 PMC4790086

[B32] Li Z. C. Jia Y. P. Wang Y. Qi J. L. Han X. P. (2018). Effects of dexmedetomidine post-treatment on BDNF and VEGF expression following cerebral ischemia/reperfusion injury in rats. Mol. Med. Rep. 17 (4), 6033–6037. 10.3892/mmr.2018.8597 29436655

[B33] Liu C. Li Z. Xi H. (2022). Bioinformatics analysis and *in vivo* validation of ferroptosis-related genes in ischemic stroke. Front. Pharmacol. 13, 940260. 10.3389/fphar.2022.940260 36506580 PMC9729703

[B34] Liu H. Wei X. Kong L. Liu X. Cheng L. Yan S. (2015). NOD2 is involved in the inflammatory response after cerebral ischemia-reperfusion injury and triggers NADPH oxidase 2-derived reactive oxygen species. Int. J. Biol. Sci. 11 (5), 525–535. 10.7150/ijbs.10927 25892960 PMC4400384

[B35] Longa E. Z. Weinstein P. R. Carlson S. Cummins R. (1989). Reversible middle cerebral artery occlusion without craniectomy in rats. stroke 20 (1), 84–91. 10.1161/01.str.20.1.84 2643202

[B36] Lu Y. Liu Y. Zhou J. Li D. Gao W. (2021). Biosynthesis, total synthesis, structural modifications, bioactivity, and mechanism of action of the quinone‐methide triterpenoid celastrol. Med. Res. Rev. 41 (2), 1022–1060. 10.1002/med.21751 33174200

[B37] Mocco J. Mack W. J. Ducruet A. F. Sosunov S. A. Sughrue M. E. Hassid B. G. (2006). Complement component C3 mediates inflammatory injury following focal cerebral ischemia. Circ. Res. 99 (2), 209–217. 10.1161/01.RES.0000232544.90675.42 16778128

[B38] Palakurti R. Biswas N. Roy S. Gnyawali S. C. Sinha M. Singh K. (2023). Inducible miR-1224 silences cerebrovascular Serpine1 and restores blood flow to the stroke-affected site of the brain. Mol. Ther. Nucleic Acids 31, 276–292. 10.1016/j.omtn.2022.12.019 36726407 PMC9868883

[B39] Paul S. Candelario-Jalil E. (2021). Emerging neuroprotective strategies for the treatment of ischemic stroke: an overview of clinical and preclinical studies. Exp. Neurol. 335, 113518. 10.1016/j.expneurol.2020.113518 33144066 PMC7869696

[B40] Peschansky V. J. Wahlestedt C. (2014). Non-coding RNAs as direct and indirect modulators of epigenetic regulation. Epigenetics 9 (1), 3–12. 10.4161/epi.27473 24739571 PMC3928183

[B41] Pu Z. Bao X. Xia S. Shao P. Xu Y. (2022). Serpine1 regulates peripheral neutrophil recruitment and acts as potential target in ischemic stroke. J. Inflamm. Res. 15, 2649–2663. 10.2147/JIR.S361072 35494316 PMC9049872

[B42] Purdy M. Obi A. Myers D. Wakefield T. (2022). P- and E-selectin in venous thrombosis and non-venous pathologies. J. Thromb. Haemost. 20 (5), 1056–1066. 10.1111/jth.15689 35243742 PMC9314977

[B43] Ren Y. Gao X. P. Liang H. Zhang H. Hu C. Y. (2020). LncRNA KCNQ1OT1 contributes to oxygen-glucose-deprivation/reoxygenation-induced injury via sponging miR-9 in cultured neurons to regulate MMP8. Exp. Mol. Pathol. 112, 104356. 10.1016/j.yexmp.2019.104356 31837324

[B44] Rodriguez-Grande B. Swana M. Nguyen L. Englezou P. Maysami S. Allan S. M. (2014). The acute-phase protein PTX3 is an essential mediator of glial scar formation and resolution of brain edema after ischemic injury. J. Cereb. Blood Flow. Metab. 34 (3), 480–488. 10.1038/jcbfm.2013.224 24346689 PMC3948128

[B45] Rodriguez-Grande B. Varghese L. Molina-Holgado F. Rajkovic O. Garlanda C. Denes A. (2015). Pentraxin 3 mediates neurogenesis and angiogenesis after cerebral ischaemia. J. Neuroinflammation 12, 15. 10.1186/s12974-014-0227-y 25616391 PMC4308938

[B46] Sharifi M. A. Wierer M. Dang T. A. Milic J. Moggio A. Sachs N. (2023). ADAMTS-7 modulates atherosclerotic plaque formation by degradation of TIMP-1. Circ. Res. 133 (8), 674–686. 10.1161/CIRCRESAHA.123.322737 37675562 PMC7615141

[B47] Shen Q. Xu G. Liu J. Wang L. Zhou Y. Yu Y. (2020). Dexmedetomidine alleviates non-ventilation associated lung injury via modulating immunology phenotypes of macrophages. Life Sci. 259, 118249. 10.1016/j.lfs.2020.118249 32798558

[B48] Shi J. Yu T. Song K. Du S. He S. Hu X. (2021a). Dexmedetomidine ameliorates endotoxin-induced acute lung injury *in vivo* and *in vitro* by preserving mitochondrial dynamic equilibrium through the HIF-1a/HO-1 signaling pathway. Redox Biol. 41, 101954. 10.1016/j.redox.2021.101954 33774474 PMC8027777

[B49] Shi Y. Yi Z. Zhao P. Xu Y. Pan P. (2021b). MicroRNA-532-5p protects against cerebral ischemia-reperfusion injury by directly targeting CXCL1. Aging (Albany NY) 13 (8), 11528–11541. 10.18632/aging.202846 33867350 PMC8109118

[B50] Shindo A. Maki T. Mandeville E. T. Liang A. C. Egawa N. Itoh K. (2016). Astrocyte-derived pentraxin 3 supports blood-brain barrier integrity under acute phase of stroke. Stroke 47 (4), 1094–1100. 10.1161/STROKEAHA.115.012133 26965847 PMC4811738

[B51] Shindo A. Takase H. Hamanaka G. Chung K. K. Mandeville E. T. Egawa N. (2021). Biphasic roles of pentraxin 3 in cerebrovascular function after white matter stroke. CNS Neurosci. Ther. 27 (1), 60–70. 10.1111/cns.13510 33314664 PMC7804900

[B52] Stowe A. M. Wacker B. K. Cravens P. D. Perfater J. L. Li M. K. Hu R. (2012). CCL2 upregulation triggers hypoxic preconditioning-induced protection from stroke. J. Neuroinflammation 9, 33. 10.1186/1742-2094-9-33 22340958 PMC3298779

[B53] Sun P. Hamblin M. H. Yin K. J. (2022). Non-coding RNAs in the regulation of blood-brain barrier functions in central nervous system disorders. Fluids Barriers CNS 19 (1), 27. 10.1186/s12987-022-00317-z 35346266 PMC8959280

[B54] Suzuki S. Brown C. M. Wise P. M. (2009). Neuroprotective effects of estrogens following ischemic stroke. Front. Neuroendocrinol. 30 (2), 201–211. 10.1016/j.yfrne.2009.04.007 19401209 PMC3672220

[B55] Tirandi A. Sgura C. Carbone F. Montecucco F. Liberale L. (2023). Inflammatory biomarkers of ischemic stroke. Intern Emerg. Med. 18 (3), 723–732. 10.1007/s11739-023-03201-2 36745280 PMC10082112

[B56] Vasudeva K. Dutta A. Munshi A. (2021). Role of lncRNAs in the development of ischemic stroke and their therapeutic potential. Mol. Neurobiol. 58 (8), 3712–3728. 10.1007/s12035-021-02359-0 33818737

[B57] Wang G. Weng Y. C. Chiang I. C. Huang Y. T. Liao Y. C. Chen Y. C. (2020b). Neutralization of lipocalin-2 diminishes stroke-reperfusion injury. Int. J. Mol. Sci. 21 (17), 6253. 10.3390/ijms21176253 32872405 PMC7503651

[B58] Wang L. Liu H. Zhang L. Wang G. Zhang M. Yu Y. (2017). Neuroprotection of dexmedetomidine against cerebral ischemia-reperfusion injury in rats: involved in inhibition of NF-κB and inflammation response. Biomol. Ther. Seoul. 25 (4), 383–389. 10.4062/biomolther.2015.180 27871154 PMC5499616

[B59] Wang L. Liu W. Zhang Y. Hu Z. Guo H. Lv J. (2020a). Dexmedetomidine had neuroprotective effects on hippocampal neuronal cells via targeting lncRNA SHNG16 mediated microRNA-10b-5p/BDNF axis. Mol. Cell. Biochem. 469 (1-2), 41–51. 10.1007/s11010-020-03726-6 32323054 PMC7244615

[B60] Wang S. W. Liu Z. Shi Z. S. (2018). Non-coding RNA in acute ischemic stroke: mechanisms, biomarkers and therapeutic targets. Cell. Transpl. 27 (12), 1763–1777. 10.1177/0963689718806818 PMC6300774 30362372

[B61] Wang Y. Q. Tang Y. F. Yang M. K. Huang X. Z. (2019). Dexmedetomidine alleviates cerebral ischemia-reperfusion injury in rats via inhibition of hypoxia-inducible factor-1α. J. Cell. Biochem. 120 (5), 7834–7844. 10.1002/jcb.28058 30456861

[B62] Wang Z. Yao M. Jiang L. Wang L. Yang Y. Wang Q. (2022). Dexmedetomidine attenuates myocardial ischemia/reperfusion-induced ferroptosis via AMPK/GSK-3β/Nrf2 axis. Biomed. Pharmacother. 154, 113572. 10.1016/j.biopha.2022.113572 35988428

[B63] Wei B. Liu W. Jin L. Guo S. Fan H. Jin F. (2022). Dexmedetomidine inhibits gasdermin D-induced pyroptosis via the PI3K/AKT/GSK3β pathway to attenuate neuroinflammation in early brain injury after subarachnoid hemorrhage in rats. Front. Cell. Neurosci. 16, 899484. 10.3389/fncel.2022.899484 35800132 PMC9253293

[B64] Wu J. Vogel T. Gao X. Lin B. Kulwin C. Chen J. (2018b). Neuroprotective effect of dexmedetomidine in a murine model of traumatic brain injury. Sci. Rep. 8 (1), 4935. 10.1038/s41598-018-23003-3 29563509 PMC5862953

[B65] Wu L. Xiong X. Wu X. Ye Y. Jian Z. Zhi Z. (2020). Targeting oxidative stress and inflammation to prevent ischemia-reperfusion injury. Front. Mol. Neurosci. 13, 28. 10.3389/fnmol.2020.00028 32194375 PMC7066113

[B66] Wu M. Y. Yiang G. T. Liao W. T. Tsai A. P. Y. Cheng Y. L. Cheng P. W. (2018a). Current mechanistic concepts in ischemia and reperfusion injury. Cell. Physiol. Biochem. 46 (4), 1650–1667. 10.1159/000489241 29694958

[B67] Xiaoqing S. Yinghua C. Xingxing Y. (2023). The autophagy in ischemic stroke: a regulatory role of non-coding-RNAs. Cell. Signal 104, 110586. 10.1016/j.cellsig.2022.110586 36608737

[B68] Yan F. Wang P. Yang X. Wang F. (2023). Long non-coding RNA HOXA11-AS regulates ischemic neuronal death by targeting miR-337-3p/YBX1 signaling pathway: protective effect of dexmedetomidine. Aging (Albany NY) 15 (7), 2797–2811. 10.18632/aging.204648 37059588 PMC10120896

[B69] Yang J. Ahn H. N. Chang M. Narasimhan P. Chan P. H. Song Y. S. (2013). Complement component 3 inhibition by an antioxidant is neuroprotective after cerebral ischemia and reperfusion in mice. J. Neurochem. 124 (4), 523–535. 10.1111/jnc.12111 23199288 PMC3557607

[B70] Yang K. Zeng L. Ge A. Wang S. Zeng J. Yuan X. (2022). A systematic review of the research progress of non-coding RNA in neuroinflammation and immune regulation in cerebral infarction/ischemia-reperfusion injury. Front. Immunol. 13, 930171. 10.3389/fimmu.2022.930171 36275741 PMC9585453

[B71] Yang P. Zhu Z. Zang Y. Bu X. Xu T. Zhong C. (2021). Increased serum complement C3 levels are associated with adverse clinical outcomes after ischemic stroke. Stroke 52 (3), 868–877. 10.1161/STROKEAHA.120.031715 33517703

[B72] Yin J. W. Li J. Ren Y. M. Li Y. Wang R. X. Wang S. (2021). Dexmedetomidine and netrin-1 combination therapy inhibits endoplasmic reticulum stress by regulating the ERK5/mef2a pathway to attenuate cerebral ischemia injury. Front. Neurosci. 15, 641345. 10.3389/fnins.2021.641345 33584197 PMC7876398

[B73] Yuan F. Fu H. Sun K. Wu S. Dong T. (2017a). Effect of dexmedetomidine on cerebral ischemia-reperfusion rats by activating mitochondrial ATP-sensitive potassium channel. Metab. Brain Dis. 32 (2), 539–546. 10.1007/s11011-016-9945-4 28035625

[B74] Yuan F. Fu H. Sun K. Wu S. Dong T. (2017b). Effect of dexmedetomidine on cerebral ischemia-reperfusion rats by activating mitochondrial ATP-sensitive potassium channel. Metab. Brain Dis. 32, 539–546. 10.1007/s11011-016-9945-4 28035625

[B75] Zhai M. Liu C. Li Y. Zhang P. Yu Z. Zhu H. (2019). Dexmedetomidine inhibits neuronal apoptosis by inducing Sigma-1 receptor signaling in cerebral ischemia-reperfusion injury. Aging (Albany NY) 11 (21), 9556–9568. 10.18632/aging.102404 31682592 PMC6874446

[B76] Zhang H. Feng Y. Si Y. Lu C. Wang J. Wang S. (2024). Shank3 ameliorates neuronal injury after cerebral ischemia/reperfusion via inhibiting oxidative stress and inflammation. Redox Biol. 69, 102983. 10.1016/j.redox.2023.102983 38064762 PMC10755590

[B77] Zhang R. Liu H. Pu L. Zhao T. Zhang S. Han K. (2023a). Global burden of ischemic stroke in young adults in 204 countries and territories. Neurology 100 (4), e422–e434. 10.1212/WNL.0000000000201467 36307222

[B78] Zhang S. Zhang Y. Zheng Y. Zhu S. Sun J. Deng Y. (2023b). Dexmedetomidine attenuates sleep deprivation-induced inhibition of hippocampal neurogenesis via VEGF-VEGFR2 signaling and inhibits neuroinflammation. Biomed. Pharmacother. 165, 115085. 10.1016/j.biopha.2023.115085 37392656

[B79] Zhao N. Xu X. Jiang Y. Gao J. Wang F. Xu X. (2019). Lipocalin-2 may produce damaging effect after cerebral ischemia by inducing astrocytes classical activation. J. Neuroinflammation 16 (1), 168. 10.1186/s12974-019-1556-7 31426811 PMC6699078

[B80] Zhao R. Y. Wei P. J. Sun X. Zhang D. H. He Q. Y. Liu J. (2023b). Role of lipocalin 2 in stroke. Neurobiol. Dis. 179, 106044. 10.1016/j.nbd.2023.106044 36804285

[B81] Zhao S. Wu W. Lin X. Shen M. Yang Z. Yu S. (2022). Protective effects of dexmedetomidine in vital organ injury: crucial roles of autophagy. Cell. Mol. Biol. Lett. 27 (1), 34. 10.1186/s11658-022-00335-7 35508984 PMC9066865

[B82] Zhao Y. Feng X. Li B. Sha J. Wang C. Yang T. (2020). Dexmedetomidine protects against lipopolysaccharide-induced acute kidney injury by enhancing autophagy through inhibition of the PI3K/AKT/mTOR pathway. Front. Pharmacol. 11, 128. 10.3389/fphar.2020.00128 32158395 PMC7052304

[B83] Zhao Y. Hua X. Ren X. Ouyang M. Chen C. Li Y. (2023a). Increasing burden of stroke in China: a systematic review and meta-analysis of prevalence, incidence, mortality, and case fatality. Int. J. Stroke 18 (3), 259–267. 10.1177/17474930221135983 36274585

