# Supplementary material for: Effect of dexmedetomidine on ncRNA and mRNA profiles of cerebral ischemia-reperfusion injury in transient middle cerebral artery occlusion rats model
Source: Front Pharmacol. 2024 Aug 7;15:1437445. doi: 10.3389/fphar.2024.1437445 (PMC11335533; doi:10.3389/fphar.2024.1437445)
Supplement: Supplementary file 1 [file DataSheet1.docx]

Supplementary Material


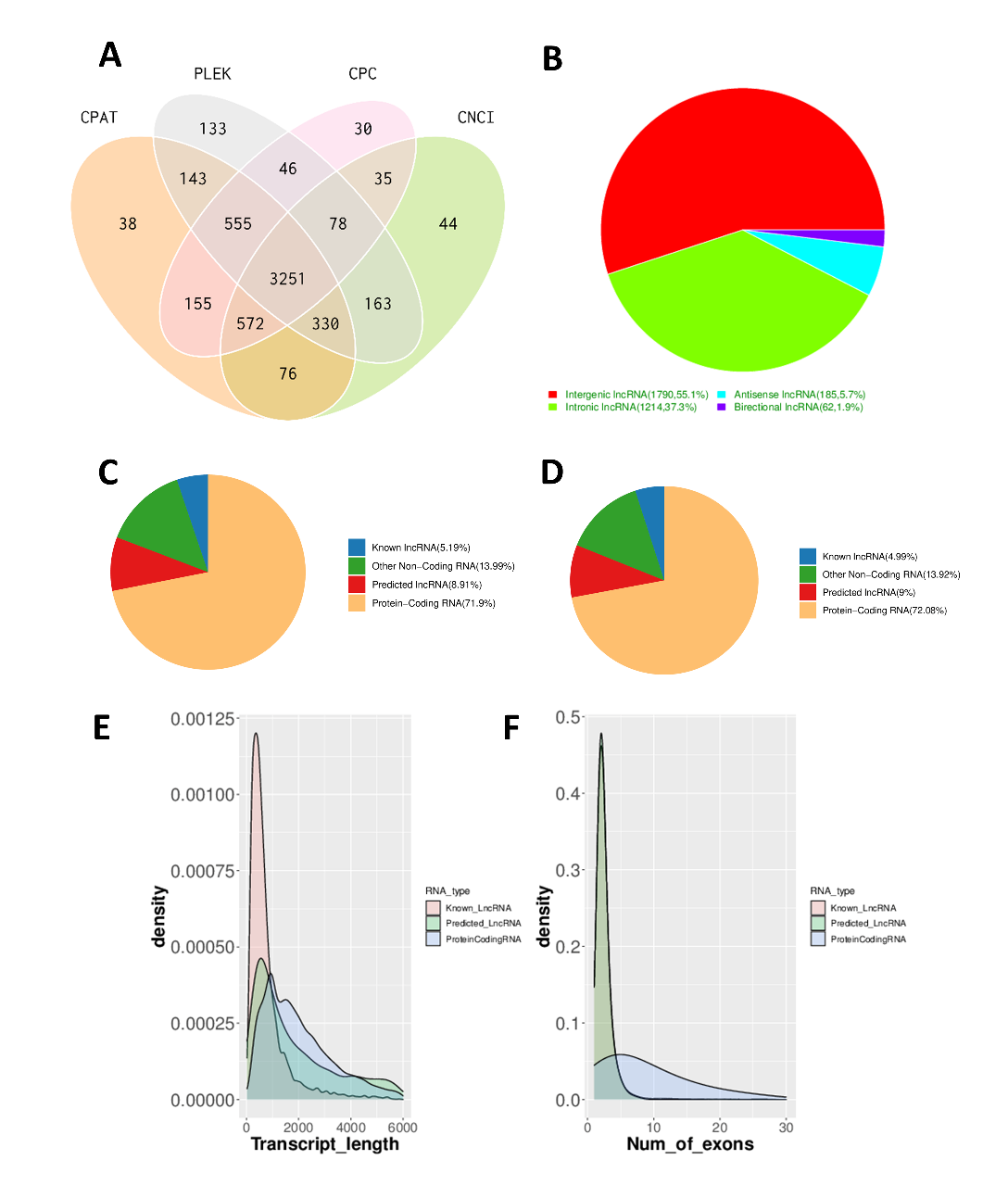


**Figure S1**: Screening and classification of the candidate long noncoding RNAs (lncRNAs) in rat cerebral cortex. (A) a Venn diagram of coding potential analysis according to strict criteria. Four tools (CPAT, PLEK, CPC, and CNCI) were used to analyze the coding potential of lncRNAs. Those simultaneously shared by four analytical tools were designated as candidate lncRNAs and used in subsequent analyses. (B). classification of the four subtypes of lncRNAs. (C, D) The proportion of protein-coding RNAs, other non-coding RNAs, know lncRNAs, and predicted lncRNAs in I/R and I/R+DEX group (E). the distribution of transcript lengths in the mRNAs and lncRNAs are displayed, the horizontal axis indicates the length of transcripts, and the vertical axis represents density. (F). The number of exons in the mRNAs and lncRNAs are displayed.

**Table S1.** Primers for RT-qPCR

| **Primer names** | **Sequences** |
| --- | --- |
| mmp3-F | ATGATGAACGATGGACAGATGA |
| mmp3-R | CATTGGCTGAGTGAAAGAGACC |
| Ntrk1-F | ACAGACTACTACCGTGTGGGA |
| Ntrk1-R | CCGAAGCTCCACACATCACT |
| MSTRG.288.3F | CCCACAGGCCAAGGGAATTT |
| MSTRG.288.3R | CCCGTGAACCCTCAACACAT |
| MSTRG.16732.1-F | CAACAATGCCCACCAACCAG |
| MSTRG.16732.1-R | CCATTACTGCCCTCCCCAAG |
| Robo2-F | TGGTCAGAGCGATCAACCCC |
| Robo2-R | AGTCGTCTTAACATGGTTTGCC |
| Chd1-F | TCCTGTTGGATTTTGCTTTAAGTT |
| Chd1-R | GCAGAAAGCTAAGCATACAGTT |

RT-qPCR, reversed transcriptase quantitative polymerase chain reaction; F, forward ; R, reverse

**TableS2**.List of up-and down-regulated mRNA, lncRNA and circRNA in I/R and I/R+DEX

|  | **I/R+ and I/R+DEX-** | **I/R- and I/R+DEX+** |
| --- | --- | --- |
| **mRNA** | **204 genes**  Tcf15,LOC100911486,Nrm,Abca16,Masp1,Retnlg,Rest,Serpinb8,Selp,Cxcl1,Bicdl2,Apln,Scin,Kitlg,Acvrl1,Klrb1a,Il23r,Orm1,Steap4,Csf3r,Knstrn,Acod1,Rbm26,Abcc4,Prrc2b,Fabp4,Cenpn,Gfi1b,Itgb7,Ccr6,Far1,C1qtnf9,Ndc80,Cxcr2,Adtrp,Gast,Il18r1,Gramd3,Mfap5,Cd2,Gnb3,Taar1,Trip4,Duox2,Cd74,Dennd2c,Fas,Pitx3,LOC100911730,Klhl30,Hcst,Gpha2,Clec4a1,Rgsl1,Lyve1,Ccl12,Sox13,Siglech,Sh2d4b,Gimap4,Dmgdh,LOC691110,Ankdd1b,Prdm14,Cd300lb,Ms4a12,Slc6a19,B4galnt3,RT1DOa,Abcc8,LOC689230,LOC691141,Lgals4,LOC297568,Stfa2,RT1Db2,Gcsam,Myo18a,Fam71a,Nfasc,Ceacam19,Ceacam16,Rufy4,Cd3e,Tbata,Adgrl4,RT1CE4,Grk6,RGD1307537,RT1Da,Ppp1r12a,Il2rg,Clec4a2,Tars,Tpcn2,Tacc2,LOC100911486,Slfn13,Tnk1,RGD1305455,LOC100362219,Cd300lf,Nckap1l,Spta1,Spry1,Fam205a,Crybb3,Snx27,LOC100360575,AABR07043711.2,NEWGENE_1589866,LOC100910252,Comp,C3,Fam124b,LOC684179,Olr1498,Igsf7,RGD1561778,RGD1559482,LOC688583,Tnfaip8l1,Oas1b,Sema4c,Zfx,Sele,Selp,Cyth4,Ppef2,Ly49si1,Relt,Dpp4,Slfn4,Esr1,Ccr2,Flot2,Cdkn1c,Milr1,Lrrc63,Ccng1,Tas1r2,Spata22,Nod2,Plec,RT1A1,Thsd1,Hyal2,Ehmt2,Adgre4,Dgat2,Tarm1,Klra2,NEWGENE_1306714,Fgfr4,AABR07000159.4,RT1Bb,Psmb8,Gkap1,Foxn3,RGD1561778,Ptdss1,Ralgds,RT1CE10,Usp1,ST7,Mrpl33,Cyp27b1,Tulp2,Vcl,Usp4,Ehmt2,Dysf,Tlr13,Soat2,Suz12,Fcgr3a,Hps1,Tmprss5,Cyp3a23/3a1,LOC498265,Gpd1,Aoah,Crispld2,NEWGENE_1305281,Ddah2,Tec,RT1A1,Eif4e,Kdsr,Bcl6b,Sec24c,Lmo7,Tead2,Rnh1,Siglec1,Mmp3,Klrk1,Tsr1,Vps52,Cfb,Tax1bp3,Hmcn1,Acod1,Zbtb20 | **163genes**  Mepe,Ivns1abp,Clcn5,Rgs9,Pld5,Nts,Cbln4,Rxrg,Trhr,Adcy8,Zfp248,Camk1g,Smyd1,Lhx8,Tac1,Kcnh7,Slc17a8,Oprk1,Reln,Igbp1b,Calb1,Tmem30b,Nkx2-1,Penk,Asb4,Zbbx,LOC500124,Slc5a7,Crhr2,Dlx5,Hcrtr2,Cpne4,Pla2g6,Nexn,Isl1,Pcdh8,Kcnq5,Ankrd34c,Gpx4,Sertm1,Ntrk1,Syt10,Fbxo39,Slc32a1,Tyr,Ttr,Epb41l3,Rfx3,Ccl21,Gnal,Sh3rf2,Htr4,Lipm,Gng7,Avp,Negr1,Tfap2b,Crabp1,LOC100909434,Gpr101,Tpte2,Scel,Usp2,Ccdc187,Lingo4,Arpp21,Magee2,Pdyn,Grem1,Nup50,Pcdh10,Cdh7,Stpg1,Hapln1,Pde10a,LOC100911951,Atp6ap1l,Drd2,Hnrnpd,Tmem132c,RGD1563714,Lrrc10b,AABR07058017.1,Gfral,Tmc5,Nalcn,Stum,Ankub1,Tbc1d14,Ccdc33,Mroh2b,Tfap2d,Slc12a5,Htr4,Fam84a,Ntng1,Gpr165,Bnc1,Mfsd4,Ankk1,Cpne7,Adam22,Col25a1,Cdk15,Ttc39a,Gpr6,Bdnf,Rad51ap2,Adcy9,Pknox2,Nlgn3,Mef2c,Mef2c,Urgcp,Lynx1,Stard8,Rbm28,Rxrg,Slc5a7,Atp2b3,Esrra,AABR07002783.1,Ank3,Kri1,Lemd1,Cacna2d2,Slc12a3,Ankrd27,Rgs12,Cep290,LOC103690166,Rbfox1,Scn5a,Eya3,Krt77,Tsga10,Cdh8,Clasp1,Ccdc39,Gabbr1,Shank3,Ptprb,Grik1,Zim1,Podn,Slc24a4,Adcy6,Taok3,Six3,Ckmt1,Penk,Exph5,Tacc2,Doc2b,Znrd1as1,Polk,Nhs,Gpam,Rsph10b,Slc17a8,Arhgap36,Dgkk, |
| **lncRNA** | **53 genes**  ENSRNOT00000077838，ENSRNOT00000078442，ENSRNOT00000080248，ENSRNOT00000080805，ENSRNOT00000082324，ENSRNOT00000085871，ENSRNOT00000089642，ENSRNOT00000089677，ENSRNOT00000089706，ENSRNOT00000091786，MSTRG.10317.2 MSTRG.10467.1, MSTRG.10721.2, MSTRG. 11683.20, MSTRG.11983.1, MSTRG.1317.1, MSTRG.13694.1, MSTRG.14028.2, MSTRG.14622.2, MSTRG.1537.109, MSTRG.1537.25, MSTRG.15674.1, MSTRG.15692.1, MSTRG.17966.6, MSTRG.19374.3, MSTRG.19416.1,MSTRG.20058.1,MSTRG.20145.3,MSTRG.20145.5,MSTRG.2024.4,MSTRG.2024.9,MSTRG.20496.5,MSTRG.20521.1,MSTRG.20765.3,MSTRG.249.1,MSTRG.275.1,MSTRG.288.2,MSTRG.288.3,MSTRG.3146.1,MSTRG.357.1,MSTRG.3884.2,MSTRG.4442.3,MSTRG.4526.2,MSTRG.4572.1,MSTRG.4573.1,MSTRG.4704.5,MSTRG.5793.4,MSTRG.6915.1,MSTRG.6952.2,MSTRG.7206.1,MSTRG.7764.4,MSTRG.862.2,MSTRG.9036.4 | **55 genes**  ENSRNOT00000076576,ENSRNOT00000080825,ENSRNOT00000088398,ENSRNOT00000092666,MSTRG.10070.6,MSTRG.1022.2,MSTRG.10552.2,MSTRG.10738.2,MSTRG.10856.1,MSTRG.10861.1,MSTRG.1128.16,MSTRG.12046.37,MSTRG.12519.1,MSTRG.12527.1,MSTRG.12545.3,MSTRG.1537.3,MSTRG.1537.67,MSTRG.1537.8,MSTRG.1537.86,MSTRG.1548.1,MSTRG.1548.2,MSTRG.1662.12,MSTRG.1662.27,MSTRG.16732.1,MSTRG.17121.39,MSTRG.18648.8,MSTRG.18701.1,MSTRG.20966.1,MSTRG.21050.1,MSTRG.21105.5,MSTRG.2395.1,MSTRG.2548.1,MSTRG.2610.3,MSTRG.2626.2,MSTRG.319.1,MSTRG.332.2,MSTRG.367.4,MSTRG.3855.9,MSTRG.4442.2,MSTRG.4704.3,MSTRG.4831.14,MSTRG.4832.11,MSTRG.6027.1,MSTRG.6457.1,MSTRG.6581.2,MSTRG.6968.1,MSTRG.8057.1,MSTRG.8776.4,MSTRG.8839.1,MSTRG.8972.4,MSTRG.9266.1,MSTRG.9268.1,MSTRG.9558.1,MSTRG.9593.1,MSTRG.9633.3, |
| **circRNA** | **902genes**  1:101799504\|101801254,1:102999437\|103001464,1:104750774\|104787042,1:114492440\|114503037,1:125151282\|125157895,1:126797300\|126824250,1:127881908\|127887913,1:128086078\|128090361,1:128501692\|128526855,1:13262070\|13280169,1:13312318\|13319919,1:134794264\|134848903,1:140044382\|140158228,1:142076493\|142079604,1:142533729\|142537799,1:154405931\|154411147,1:156552340\|157125505,1:162830920\|162843974,1:163564635\|163598771,1:165340205\|165354415,1:165366392\|165367044,1:167248618\|167249761,1:167292202\|167293396,1:1720412\|1723417,1:17549813\|17560520,1:176380041\|176382073,1:185263914\|185291713,1:185902388\|186009525,1:185970229\|186009525,1:186067922\|186114868,1:187803376\|187812018,1:195078078\|195087611,1:199846913\|199849967,1:204975105\|204988430,1:20961258\|20962540,1:216371821\|216382773,1:219119473\|219122692,1:219981557\|219981979,1:221822465\|221829297,1:222318743\|222319656,1:23430279\|23458064,1:235711982\|235737635,1:235763930\|235780409,1:235777942\|235780409,1:242244899\|242248135,1:245955041\|246031901,1:247711959\|247736943,1:25216049\|25284394,1:255685048\|255774681,1:25767968\|25787662,1:259396273\|259479455,1:260774582\|260791888,1:260893829\|260898687,1:261198164\|261200060,1:263873195\|263879968,1:265714699\|265717792,1:268601688\|268611753,1:27476375\|27562055,1:277952138\|277997007,1:278698312\|278900769,1:280167086\|280184520,1:282151347\|282155817,1:282194100\|282198076,1:282225916\|282231487,1:32262696\|32264516,1:3919308\|3943683,1:43594671\|43617390,1:43823612\|43831931,1:44366948\|44372134,1:44366948\|44393411,1:57709952\|57712920,1:59312916\|59339853,1:59322438\|59339853,1:6521963\|6546490,1:6534185\|6575208,1:72718573\|72718974,1:72975360\|72980019,1:82213523\|82214415,1:82517496\|82538202,1:89001745\|89004978,1:89353098\|89355401,1:90116534\|90126552,1:91884973\|91890053,10:104140408\|104141055,10:105887011\|105890450,10:10849180\|10852471,10:109013149\|109018917,10:109310593\|109311350,10:109574583\|109588126,10:110727632\|110760448,10:13460955\|13479320,10:1484236\|1485251,10:15544776\|15555433,10:15989960\|15998622,10:3260919\|3275794,10:3272651\|3296133,10:35816390\|35818308,10:37194412\|37194876,10:39813195\|39833654,10:40019676\|40035311,10:4140144\|4172981,10:43591327\|43596422,10:48015493\|48037423,10:48452001\|48528848,10:48683733\|48696065,10:48694567\|48705799,10:50527421\|50562722,10:550482\|578622,10:55354861\|55362785,10:56244288\|56246877,10:574395\|593608,10:578446\|593608,10:58739752\|58771849,10:58762060\|58771849,10:61230757\|61236946,10:61467902\|61483913,10:61467902\|61498597,10:61639917\|61658848,10:64829256\|64831635,10:66779889\|66789207,10:66785840\|66786996,10:66806816\|66822005,10:66814999\|66890392,10:66889960\|66894799,10:67539765\|67549049,10:67579952\|67587851,10:67741790\|67742140,10:71266379\|71298197,10:71399723\|71408228,10:71567498\|71577610,10:72329605\|72340184,10:72856016\|73038307,10:73615333\|73627965,10:73759988\|73778454,10:74555144\|74562163,10:78063351\|78073611,10:78181489\|78191113,10:81004829\|81193040,10:83101669\|83114195,10:85164768\|85171060,10:85166708\|85171060,10:86199507\|86200782,10:88397108\|88397582,10:89946303\|89956464,10:92349223\|92364113,10:93451477\|93483645,10:94362934\|94364773,10:95288570\|95299099,10:98339552\|98360556,11:20474484\|20638592,11:20531606\|20650229,11:24447169\|24480063,11:24527899\|24589526,11:31557946\|31569614,11:31646417\|31660183,11:34060062\|34066034,11:34613499\|34621503,11:34879058\|34922336,11:4286079\|4327130,11:51068884\|51110751,11:51077792\|51088607,11:54412416\|54423219,11:54416332\|54425138,11:66406507\|66426089,11:677727\|678387,11:70810417\|70820621,11:72210571\|72236448,11:73151999\|73179042,11:73974471\|73987564,11:84521876\|84522603,11:87575925\|87577497,11:87900974\|87902915,11:88753588\|88762839,11:89328437\|89332868,12:12898458\|12907771,12:13526520\|13527329,12:2182120\|2188197,12:23962947\|23968609,12:25442325\|25460812,12:30480765\|30486660,12:37500716\|37507430,12:37605184\|37625215,12:37728811\|37753501,12:37741418\|37761839,12:37757101\|37760713,12:38080067\|38084011,12:38375308\|38400646,12:38399702\|38412118,12:39586153\|39591110,12:40269634\|40270510,12:46341919\|46414710,12:47225890\|47228816,12:49699399\|49724273,12:51981500\|51991906,12:52140598\|52152750,12:6938605\|6952864,12:6941069\|6952864,12:775678\|780793,13:106613914\|106617947,13:109403515\|109418235,13:109403515\|109459916,13:110443909\|110447458,13:110447185\|110450689,13:113828023\|113844645,13:22412187\|22444244,13:25565372\|25569449,13:25747905\|25748576,13:25822695\|25823457,13:26340136\|26349774,13:35585251\|35608096,13:35704642\|35719842,13:35769419\|35822951,13:44883082\|44896587,13:44898820\|44911389,13:51442604\|51450355,13:51708289\|51714375,13:53167593\|53179800,13:56151434\|56173172,13:69355877\|69385072,13:70261820\|70263383,13:73554326\|73569156,13:73615097\|73615713,13:74298540\|74331212,13:78983041\|78991952,13:83602188\|83620518,13:86424477\|86436725,13:88478383\|88480700,13:89107668\|89176307,13:98068180\|98075073,13:98311826\|98423223,14:104355789\|104364850,14:108157937\|108189729,14:108189529\|108203293,14:108331019\|108358009,14:108331019\|108374435,14:108347874\|108367329,14:108357849\|108367329,14:110683107\|110734538,14:113271542\|113282291,14:113910057\|113928058,14:113951426\|113958155,14:114582964\|114649217,14:115183821\|115195697,14:14574448\|14645391,14:14986610\|14990106,14:15091390\|15127296,14:15091390\|15141031,14:23524353\|23529139,14:25791641\|25796293,14:25859208\|25865192,14:25859208\|25911503,14:33075896\|33089673,14:39169609\|39186197,14:4178531\|4197781,14:43420836\|43461320,14:43556760\|43581074,14:44221926\|44234560,14:7291691\|7293326,14:79953865\|79960727,14:81534519\|81598570,14:83260146\|83262412,14:83919054\|83924540,14:85011274\|85057435,14:9226094\|9247429,14:92484936\|92487269,14:9289221\|9290346,15:1005261\|1011558,15:103737778\|103747183,15:107951764\|107952407,15:110546595\|110550352,15:15499315\|15504530,15:19638868\|19643375,15:24361728\|24363134,15:25298949\|25344392,15:25897651\|25918193,15:25909344\|25920532,15:25917999\|25921639,15:37056224\|37056934,15:37740016\|37762996,15:39715651\|39725690,15:39793373\|39797194,15:41086581\|41104188,15:4146307\|4173486,15:4152545\|4162016,15:43501489\|43539555,15:47466601\|47473249,15:48443441\|48446043,15:59233127\|59250976,15:60819559\|60847217,15:60892679\|60895084,15:61646233\|61651842,15:70121769\|70139170,15:837322\|887290,15:8920740\|8979459,15:8920740\|9086278,15:93656643\|93663365,15:93739958\|93741147,16:20604417\|20606895,16:2117271\|2179241,16:23687864\|23729219,16:3068022\|3072642,16:40469880\|40483041,16:4269443\|4283627,16:46873084\|46876213,16:48960409\|48962854,16:50413024\|50413854,16:54048948\|54107348,16:54080267\|54104342,16:62509443\|62530689,16:7107281\|7139660,16:73966235\|73978345,16:76018333\|76070467,16:81105465\|81114000,17:10096739\|10107746,17:16124900\|16153210,17:18304439\|18311110,17:19160994\|19219406,17:27438853\|27449400,17:34704621\|34709866,17:43258446\|43270450,17:43258446\|43284309,17:48371361\|48399245,17:5232517\|5234506,17:5238037\|5254729,17:53528580\|53542696,17:54035369\|54048283,17:5429865\|5441885,17:5513194\|5571375,17:5563519\|5591590,17:62358469\|62375850,17:62358469\|62388780,17:63635093\|63657910,17:63691207\|63716786,17:65826669\|65837282,17:65826669\|65857795,17:65832400\|65857795,17:76212065\|76265943,17:76360839\|76383098,17:77607620\|77613583,17:83375370\|83420919,17:84888802\|84904355,17:84904260\|84940529,17:87622185\|87647147,17:87658633\|87664734,17:88451147\|88506410,17:89213534\|89229807,18:1185270\|1203177,18:14595550\|14643454,18:15347145\|15359840,18:15480332\|15494701,18:17218299\|17258865,18:17404077\|17409355,18:24470544\|24478015,18:25645682\|25645954,18:26584221\|26613097,18:26633551\|26660117,18:27054582\|27086614,18:27242909\|27250440,18:27701786\|27708817,18:28026848\|28028197,18:28472290\|28490227,18:31605777\|31610800,18:3191101\|3206664,18:4246730\|4265124,18:4252920\|4265124,18:4257944\|4265124,18:44519513\|44532874,18:48284142\|48300219,18:48680858\|48700303,18:49891333\|49891588,18:51560416\|51574789,18:5158060\|5194413,18:53094773\|53099480,18:55960998\|55966549,18:59245089\|59283798,18:59752584\|59753141,18:59946985\|59964492,18:60639860\|60654344,18:63440362\|63465095,18:63618420\|63621400,18:63630085\|63642688,18:66597053\|66645730,18:69634006\|69647714,18:69742979\|69761289,18:70254197\|70255206,18:72571325\|72571612,18:72571325\|72597936,18:75203861\|75263369,18:77447512\|77489468,18:8179579\|8190394,19:12485093\|12491559,19:2506536\|2521388,19:25669577\|25722171,19:32470120\|32472456,19:33058446\|33073342,19:34227324\|34241861,19:34346074\|34395489,19:39176540\|39187048,19:41322174\|41349686,19:43053195\|43061646,19:43207756\|43209301,19:45073283\|45089680,19:46552916\|46559348,19:52410334\|52417467,19:54786578\|54810657,19:56684651\|56690330,19:58759728\|58764349,19:60322405\|60332725,19:742482\|745658,19:9722490\|9728783,19:9727305\|9728783,2:104882050\|104892419,2:111537046\|111772738,2:117094733\|117103679,2:119285165\|119311765,2:119374436\|119405809,2:123698202\|123723116,2:139466768\|139520450,2:140582486\|140607346,2:144849839\|144862593,2:150838385\|150858459,2:157334132\|157335656,2:165687204\|165704601,2:166406958\|166423542,2:173488961\|173495892,2:174065229\|174074420,2:177878125\|177885499,2:178246797\|178249289,2:181861643\|181864506,2:183176318\|183179196,2:18525795\|18531203,2:185612008\|185635510,2:185908549\|185967904,2:188301023\|188348762,2:188337238\|188348762,2:195665453\|195668745,2:196198427\|196202153,2:196608497\|196623842,2:196608497\|196628987,2:196637067\|196642711,2:198843570\|198850683,2:200003303\|200022431,2:205814398\|205838582,2:206260840\|206264944,2:207356978\|207360278,2:212427056\|212429470,2:21828582\|21921128,2:221062375\|221072200,2:224794357\|224798932,2:225570720\|225578515,2:22775246\|22782386,2:23068950\|23072842,2:231056605\|231078948,2:23399715\|23483987,2:236621881\|236628051,2:237794153\|237799015,2:238162944\|238246933,2:240685748\|240701716,2:248779402\|248789501,2:2494599\|2505817,2:25087997\|25091646,2:252274389\|252280201,2:252803944\|252832870,2:257589919\|257599468,2:257739283\|257854741,2:25870923\|25909703,2:258825484\|258830770,2:25906768\|25927179,2:264798623\|264809264,2:264871216\|264883953,2:266157333\|266160493,2:29697478\|29700175,2:30651173\|30663061,2:33818119\|33826241,2:33935271\|33955969,2:33937182\|33955969,2:33950934\|33952806,2:34315163\|34331310,2:39028857\|39042852,2:4143950\|4174344,2:43015731\|43018584,2:43046038\|43068677,2:44749022\|44756514,2:45801786\|45813991,2:46389859\|46438887,2:57334852\|57471103,2:9245768\|9261301,20:14319170\|14324124,20:14342249\|14361700,20:14440779\|14461521,20:15105444\|15144660,20:15157024\|15202495,20:1976113\|1976812,20:28087102\|28110280,20:28662866\|28682655,20:44909953\|44927561,20:46629859\|46634395,20:48038697\|48119235,20:50725400\|50751445,20:5090501\|5090805,20:54207315\|54269470,20:54260924\|54269470,20:55285814\|55286373,20:5549085\|5550879,20:5851258\|5853847,20:7549783\|7573740,20:8291482\|8304555,20:9207480\|9213292,3:110880970\|110891998,3:111234984\|111242797,3:111728536\|111730097,3:112699901\|112728973,3:112808478\|112820089,3:112820022\|112830807,3:112925889\|112931707,3:113172295\|113186152,3:113218951\|113222156,3:11325952\|11328601,3:11833194\|11837525,3:119394452\|119397247,3:121519927\|121554024,3:122130941\|122136862,3:123249654\|123256943,3:123326060\|123346073,3:123825681\|123833222,3:124668363\|124673546,3:128236234\|128272114,3:129146876\|129229202,3:13513288\|13602354,3:136701840\|136736403,3:136874645\|136896550,3:141240315\|141270969,3:150005719\|150014299,3:15225636\|15239708,3:152335580\|152346695,3:153403008\|153410702,3:154521486\|154533322,3:162506117\|162536454,3:162763508\|162770832,3:163397320\|163411447,3:164945234\|164976288,3:167989369\|167999762,3:175323014\|175327679,3:175512077\|175527973,3:176829663\|176842301,3:1784562\|1789155,3:2021173\|2037071,3:2021173\|2037656,3:2025861\|2037656,3:21850499\|21860492,3:25904155\|25905177,3:25991780\|26025779,3:29590033\|29660977,3:33168811\|33364916,3:33465220\|33467909,3:3403016\|3414759,3:38233588\|38254161,3:38559912\|38614592,3:47635223\|47639457,3:48204874\|48217321,3:48801236\|48815130,3:51145768\|51149884,3:54455979\|54519757,3:56215576\|56229714,3:58050908\|58067040,3:58893605\|58908018,3:60026746\|60032771,3:63416257\|63433816,3:64062990\|64119899,3:64312162\|64362882,3:66824713\|66827909,3:66857281\|66885086,3:79504081\|79505361,3:79514711\|79521792,3:80086313\|80091534,3:82700062\|82725485,3:8547346\|8577209,3:91713775\|91769029,3:93971370\|93982352,3:94142096\|94152694,3:94360855\|94363073,3:94990702\|94996833,3:97727432\|97727679,3:9865677\|9887262,4:10225077\|10233323,4:108696485\|108701068,4:113743666\|113745993,4:115631196\|115670939,4:118486088\|118503935,4:118817454\|118832427,4:119055923\|119083375,4:124800135\|124807181,4:125785761\|125808379,4:132079305\|132094750,4:133073964\|133094100,4:140470174\|140505698,4:140870957\|140872418,4:151418063\|151428896,4:151418551\|151428896,4:152526403\|152529177,4:155716221\|155718114,4:160036663\|160046029,4:160275564\|160293758,4:169005310\|169016804,4:169998117\|169998594,4:174720692\|174755822,4:174816236\|174829004,4:177272028\|177281107,4:177280993\|177297215,4:179275043\|179298028,4:180610928\|180613165,4:182648931\|182826795,4:182811463\|182826795,4:18311528\|18331379,4:21535264\|21541936,4:2557720\|2564215,4:27206626\|27209887,4:28458758\|28478841,4:28474639\|28495139,4:28474639\|28514174,4:38304781\|38338391,4:50352168\|50417163,4:50451125\|50459347,4:58679484\|58748928,4:58748816\|58780524,4:61439661\|61482074,4:6237397\|6244310,4:66172555\|66195339,4:66207490\|66214585,4:66888427\|66895663,4:67463557\|67483807,4:84692534\|84698509,4:86413264\|86417288,4:87186852\|87188712,4:95892497\|95896375,4:99970321\|99989565,5:106607472\|106654204,5:106654112\|106684153,5:113599521\|113632712,5:117210705\|117257388,5:117742776\|117754533,5:122655270\|122671094,5:122655270\|122683766,5:122659399\|122678330,5:127435828\|127447700,5:128096605\|128118972,5:129413066\|129528945,5:129448885\|129457884,5:129585577\|129647021,5:135596270\|135626872,5:136143122\|136154645,5:139394727\|139421497,5:139800834\|139816856,5:141149663\|141162457,5:14305876\|14333815,5:144534955\|144547479,5:147859728\|147861952,5:148835226\|148881293,5:151371115\|151377439,5:151509414\|151519907,5:153221455\|153228502,5:154352340\|154354091,5:156478182\|156484234,5:156511839\|156517254,5:157863697\|157864867,5:157912910\|157922337,5:160037224\|160043135,5:166023283\|166030168,5:167455211\|167486126,5:167547693\|167615430,5:173203485\|173204284,5:21277740\|21344908,5:21830878\|21832691,5:24303433\|24314713,5:24410842\|24411969,5:28538337\|28557266,5:43610593\|43611262,5:48026297\|48029818,5:48583539\|48636636,5:57335882\|57336829,5:58045598\|58070135,5:59619932\|59624459,5:60601995\|60606533,5:64346707\|64352506,5:70292288\|70369574,5:70552374\|70557544,5:71790897\|71792149,5:72119820\|72124022,5:73949537\|73976112,5:75979765\|75996811,5:76562980\|76576806,5:7830490\|7855124,5:7839835\|7855124,5:92935424\|92995720,5:92983736\|93050809,5:99483515\|99505496,6:100337225\|100434945,6:103527570\|103553352,6:104309774\|104313679,6:105726238\|105734728,6:107180835\|107195819,6:109742370\|109800905,6:109764831\|109875835,6:109817920\|109875835,6:112684486\|112984111,6:115064735\|115117832,6:115092099\|115138837,6:124463025\|124505972,6:124579563\|124629605,6:133761889\|133773274,6:134913910\|134933399,6:15082851\|15085804,6:21771178\|21797140,6:21815385\|21827554,6:21953284\|21974221,6:21981543\|21984543,6:22247121\|22255740,6:23236297\|23291583,6:23460178\|23465053,6:23557202\|23563843,6:26074557\|26099044,6:26081724\|26099044,6:28769634\|28785741,6:3333439\|3345418,6:38426835\|38441192,6:38515315\|38578509,6:38730920\|38762910,6:43306391\|43333304,6:45526457\|45537399,6:53651726\|53732376,6:55920976\|56036516,6:57720453\|57821418,6:57761113\|57821418,6:57821344\|57839749,6:60570821\|60618957,6:60660400\|60700690,6:64794456\|64810755,6:65129296\|65257787,6:6989418\|7017286,6:73769152\|73868895,6:73787676\|73854960,6:73868775\|73908680,6:75639848\|75654440,6:80306621\|80307306,6:828602\|940219,6:86842054\|86843095,6:86842054\|86849086,6:872807\|907083,6:91552253\|91568286,6:91669616\|91674153,6:91912886\|91945956,6:91942178\|91980480,6:97926551\|97977520,7:104718591\|104749401,7:104731318\|104800536,7:104741465\|10475494,7:116700240\|116701695,7:123052952\|123055378,7:12596464\|12598185,7:126265888\|126271959,7:129690245\|129708614,7:137274286\|137281978,7:137770661\|137784670,7:140828965\|140845680,7:141855875\|141856914,7:142153995\|142158382,7:2440628\|2448053,7:24777879\|24809425,7:26965506\|26969212,7:29361394\|29363237,7:30929783\|31374263,7:31194110\|31196403,7:3268770\|3273254,7:34057078\|34058440,7:41322521\|41326655,7:52310713\|52350037,7:57341120\|57434518,7:59955208\|59957230,7:60790156\|60795512,7:64024698\|64033477,7:66761911\|66763153,7:80415733\|80428397,7:80721364\|80750790,7:90253169\|90277429,7:97990433\|98009848,7:98792746\|98802216,8:103783872\|103790256,8:104199588\|104202285,8:107327241\|107363541,8:109230279\|109254764,8:115779975\|115788488,8:118860285\|118873176,8:120435062\|120450333,8:122129371\|122142063,8:123285546\|123290769,8:126419372\|126423244,8:127223287\|127243078,8:19875053\|19890764,8:23508992\|23524947,8:25620092\|25651193,8:29944044\|29945357,8:32487066\|32489431,8:36271272\|36274775,8:42448061\|42459487,8:46851696\|46916766,8:46943246\|46963285,8:48538280\|48538527,8:49136262\|49139022,8:52127636\|52153454,8:58040119\|58083723,8:60441497\|60521742,8:60493750\|60579877,8:60917720\|60920949,8:61768727\|61769811,8:64130841\|64133533,8:64610881\|64624646,8:64610881\|64641748,8:67841243\|67841687,8:69971772\|69979657,8:70331097\|70347005,8:71601458\|71601850,8:77136036\|77204153,8:77192321\|77196513,8:79330088\|79338690,8:79675935\|79680299,8:82038973\|82065175,8:82442554\|82443621,8:84741970\|84748991,8:96046333\|96073130,9:100575892\|100588953,9:10476177\|10480001,9:10704605\|10710650,9:111607928\|111653766,9:11187797\|11209094,9:112408205\|112436745,9:113414837\|113452381,9:119126801\|119148487,9:119750465\|119771286,9:119750465\|119792090,9:1442054\|1545372,9:16959148\|16965491,9:18319246\|18326823,9:19451520\|19452868,9:19537850\|19540863,9:20794681\|20811799,9:28529457\|28558845,9:28552021\|28732920,9:28602646\|28609952,9:31657373\|31711428,9:42920694\|42924023,9:43980214\|43994897,9:45203455\|45205627,9:53475012\|53508890,9:54594197\|54648028,9:54647924\|54672875,9:63508957\|63636231,9:63534189\|63614711,9:64754127\|64821531,9:66758471\|66774536,9:66793691\|66796534,9:66796280\|66804771,9:66885432\|66915168,9:67242046\|67269326,9:7001357\|7002330,9:71836397\|71850536,9:71954951\|71960153,9:73416480\|73437304,9:73497828\|73511373,9:75258833\|75299016,9:81734340\|81746224,9:81734340\|81749099,9:83141975\|83144611,9:86060694\|86069833,9:88630024\|88642370,9:90884970\|90891953,9:92333396\|92336862,9:95043833\|95049890,9:96277398\|96279751,9:96804834\|96866552,X:115017927\|115022232,X:128233906\|128251371,X:135964894\|136050917,X:153570079\|153586133,X:15530391\|15532829,X:158876005\|158885745,X:22280471\|22291408,X:32346665\|32353152,X:33946310\|34037071,X:37539696\|37556244,X:38409898\|38449726,X:39751868\|39800248,X:4819220\|4837324,X:52214437\|52262950,X:55460566\|55497076,X:76924257\|76961132,X:79859828\|79882917,X:82932968\|82946086,X:84678296\|84714373,X:984901\|990236,X:99852198\|99926471,Y:1037808\|1128138,Y:523566\|532697 | **183genes**  1:102613968\|102645633,1:121403792\|121409783,1:140131618\|140158228,1:146456005\|146510287,1:174527904\|174529818,1:175225210\|175284224,1:185722677\|185902595,1:235720725\|235771816,1:240355152\|240449390,1:255956584\|255976428,1:255970949\|255976428,1:278656261\|278731755,1:52377326\|52401374,1:56902969\|56915732,1:73746711\|73747791,10:18224064\|18250399,10:1910189\|1934477,10:35898838\|35901897,10:52216060\|52270583,10:55419844\|55436069,10:57517680\|57541484,10:61207781\|61217982,11:10020678\|10068759,11:10106740\|10110668,11:11103972\|11139578,11:15461115\|15474826,11:24479964\|24550752,11:47157561\|47162537,11:51049296\|51068978,11:71649617\|71658165,11:72798898\|72814254,11:73747153\|73759575,12:10499000\|10515204,12:40652886\|40665373,12:51582921\|51592889,13:109403515\|109442614,13:69936554\|69944404,13:74485162\|74513464,13:95098506\|95348984,14:115198980\|115237537,14:12003226\|12005652,14:25899869\|25945016,14:25909290\|25945016,14:28418126\|28495950,14:38195749\|38206899,14:39730079\|39755962,14:4154610\|4182331,15:109943076\|109970339,15:25905491\|25921639,15:2965953\|2966833,15:37714720\|37725296,15:37860371\|37863795,15:4124785\|4129118,15:48278041\|48284550,15:61664226\|61678600,16:20913083\|20916267,16:49367616\|49383867,16:54057183\|54100069,16:56297404\|56350456,16:71097098\|71104753,16:71192886\|71203611,16:71853351\|71865681,16:78565538\|78613084,17:10096739\|10124159,17:27571479\|27602936,17:58006111\|58023915,17:718534\|739440,17:83567831\|83589045,17:9912158\|9929565,18:1203074\|1210713,18:13369444\|13428569,18:17224244\|17258865,18:17300168\|17320717,18:28856909\|28871848,18:32047892\|32094303,18:57743427\|57755793,18:59190041\|59245202,18:81170024\|81428974,19:16955422\|16972431,19:24181887\|24197730,19:34233020\|34346224,19:41745234\|41752386,19:55882755\|55896592,2:128562842\|128590589,2:128619074\|128668115,2:140496833\|140515786,2:147877035\|148009588,2:183520240\|183555315,2:185612008\|185612494,2:185908549\|185922150,2:186232196\|186232527,2:188127807\|188128630,2:189721672\|189729005,2:205814398\|205825204,2:219620159\|219626341,2:258836959\|258932200,2:27441735\|27447367,2:3809213\|3839676,20:14358035\|14375435,20:47806643\|47896656,3:104980955\|105017214,3:12103051\|12115233,3:12860437\|12886616,3:147780064\|147785568,3:21844719\|21904269,3:23488872\|23493868,3:25875450\|25905177,3:48106099\|48130852,3:54455979\|54463443,3:62681497\|62688363,3:93705174\|93733686,4:131403367\|131453028,4:174720692\|174742961,4:2737747\|2748632,4:27509136\|27557897,4:42996228\|43010601,4:48907519\|48928205,4:50555306\|50635606,4:58143324\|58147448,4:67449579\|67455064,4:75325651\|75391362,5:117586102\|117592209,5:128130304\|128136348,5:129601063\|129647021,5:135354650\|135359472,5:14333711\|14350989,5:144939820\|144942704,5:146485000\|146526210,5:146493984\|146526210,5:147366626\|147368505,5:148790779\|148836445,5:148790779\|148847692,5:150728264\|150735555,5:156463243\|156484234,5:160244885\|160262721,5:165081766\|165083490,5:167526171\|167581074,5:167547693\|167581074,5:27386153\|27397113,5:48583539\|48623756,5:75975497\|75990306,5:75992495\|76011558,5:76711755\|76753032,6:106068325\|106097188,6:111072969\|111081661,6:111098515\|111106551,6:125776335\|125791354,6:126667976\|126671091,6:129629703\|129632684,6:145431315\|145439120,6:51145033\|51155100,6:76616895\|76626199,6:88717420\|88757918,6:9361268\|9434931,7:14246803\|14256219,7:30418294\|30439355,7:32922004\|32955235,7:52377077\|52380182,7:78347306\|78404200,7:80725042\|80745256,8:117323994\|117325025,8:11889162\|11916730,8:122760377\|122764541,8:130792978\|130799651,8:36661012\|36662130,8:58968390\|58970397,8:63689729\|63707726,8:69789523\|69795155,8:70535555\|70547228,8:71379297\|71497659,8:77136036\|77169140,9:111597035\|111618959,9:11217254\|11219449,9:15732312\|15789416,9:17166589\|17172409,9:28502814\|28609952,9:31734736\|31767934,9:7022983\|7066705,9:92360876\|92382401,X:128186178\|128198293,X:134649467\|134713998,X:39830728\|39864346,X:56083435\|56257934 |

**Table S3.** DE mRNA related to stroke in I/R group

| **Up（259）** | **Down (82)** | |
| --- | --- | --- |
| Ccl2,C3,Cxcl1,Serpine1,Hspa1a,Il6,Hspa1b,Selp,Adamts7,Ccl3,Csf3,Ptx3,Mmp8,Lif,Lcn2,Itgb3,Spp1,Lgals3,Mmp12,Gdf15,Fcgr3a,Cd14,Timp1,Hmox1,Sell,Itga2,Il1b,Sele,Mmp3,Abcc8,Kng1,Mmp13,Il1rn,Cyp1b1,Gja5,F5,Il4r,Tspo,Icam1,Lox,Ptgis,Cd79a,Adm,Tnfrsf12a,Fos,Procr,Atg9b,Olr1,Ccr2,Angpt2,Col4a1,Thbs1,Pdgfa,Igfbp3,Nos3,Nes,Mpl,Wnk1,Fabp4,Mmp9,Csf3r,Flnc,Cp,Mgp,Gnb3,Cxcr4,F7,Tnf,Itgb2,Cybb,Col4a2,Ampd1,Calca,Gja4,Scnn1a,Tnfrsf1a,Nod2,Flna,Il2ra,Tgfb1,Cyba,Thbd,Nphs2,Cdh5,Il1r1,Eng,Vdr,Gfap,Nppb,Il18r1,Fbn1,Tnfrsf1b,Bmp7,Havcr1,Cd63,Esr2,Gata4,Pla2g2a,Elane,Cdkn2b,Fas,Myd88,F10,Myl4,Pappa,Il10,Ncf1,Socs1,Ece1,Casp1,Col3a1,Park7,Stat3,Vasp,Myh9,Vwf,Kdr,Tf,Tlr2,Mmp2,C1r,Cd36,Adipoq,Ccm2,Mpo,Ephb4,Edn1,Proc,Ednra,Csf2,Ppard,Mat1a,Xdh,Lipc,Epas1,Nampt,Lmna,Agxt,Alox5ap,Ace,Ptpn22,Ucp2,Samhd1,Plat,Tbxas1,Csrp3,Tgfbr2,Pf4,Cd40,Il1a,Esr1,Mfap5,Pxdn,Slc19a1,Igf2bp2,Serpinf2,Pdgfb,C4a,Gp1ba,Il18,Slc2a1,Ldlr,Chi3l1,Ptgs2,Fn1,Slc25a24,Hbb,Ren,C4b,Calcrl,Nfe2l2,Pmf1,Cox4i2,Tek,Palld,Itgb1,Lepr,Eln,Cfh,Shmt1,Pla2g7,Tlr4,Ptgs1,Hp,Arg1,Col1a1,Gstp1,Serpinc1,Grn,Aplnr,Prkch,Ctsb,Cstb,Nde1,Nlrp3,Il6r,Cav1,Flt1,Pon3,Pdgfrb,Npy,Fgf21,Mbp,Ifih1,Pde3a,Jag1,Notch2,Col5a1,Sod2,Rbpj,Tfpi,Tp53,Actb,Notch1,Itih4,F13a1,Coq8b,Adam17,Bcl2l1,Rnf213,Vegfa,Gdnf,Notch3,Ada,Tcn2,B2m,Acvrl,,Hif1a,,MtCo1,,Apoa1,Apoe,Itga2b,Abcc9,Entpd1,Tgfbr1,Tshr,Enpp1,Apln,MtCyb,Timp3,Sh2b3,Plod1,Bcl2,Kitlg,Ggcx,Aoc3,Ngf,Tgfb3,Ednrb,MtNd1,Vhl,Asl,Ass1,Dguok,Twist1,Gtpbp3,Col5a2,Col4a4,Acta2 | Nr4a2,Ache,Mapt,Atp1a3,Adrb1,Kcnh2,Stim1,Tubb3,Sort1,Fndc5,Gria3,Casp9,Igf1,Foxp2,Tmem106b,Bdnf,Wnk3,Atp1b1,Uchl1,Alb,Kcnma1,Slc1a2,Phactr1,Dnah8,Nefl,Atrx,Mc4r,Tac1,Gpr22,Atp1a2,Cbs,Grin2b,Hdac9,Proz,Tnni3,Syp,Wfs1,Cacna1c,Scn8a,Fbxl4,Ltc4s,Srebf1,Cyp11b2,Serpini1,Jph3,Scn3b,Asph,Grin2a,Chat,Dcx,Gp6,Map2,Pvalb,Plg,Kcnj3,Htr2a,Myh7,Adrb3,Gcg,Slc19a3,P2ry12,Scn2a,Rbfox3,Kcna5,Scn1a,Scn2b,Ccr5,Htr1a,Gdf10,Agt,Kalrn,Ttr,Glp1r,Scn5a,Agtr2,Nts,Scn4b,Avp,Ntrk1,Nkx2-1,Scn1b,Shank3 |  |

**Table: S4:** Overlapped genes between Inflammation and oxidative stress-related genes

|  | **Up & down DE mRNA** |
| --- | --- |
| **Inflammation** | Orm1, Ccr6, Cxcr2, Gast, Comp, C3, Dpp4, Ccr2, Psmb8, Hps1, Reln, Ntrk1, Selp, Cxcl1, Fas, Sele, Esr1, Nod2, Mmp3, Tac1, Ttr, Bdnf |
| **oxidative stress** | Vcl, Ddah2, Crhr2, Gpx4, Tyr, Drd2, Selp, Cxcl1, Fas, Sele, Esr1, Nod2, Mmp3, Tac1, Ttr, Bdnf |
| **Inflammation & oxidative stress** | Selp, Cxcl1, Fas, Sele, Esr1, Nod2,Mmp3,Tac1,Ttr, Bdnf |

**Table S5**: Overlapped genes between stroke and cognitive disorder/sleep disorders related genes

| **IR vs S** | |
| --- | --- |
| **cognitive disorder** | **30 genes**  Asph,Procr,Csrp3,Aoc3,Gtpbp3,Adam17,Plat,Phactr1,Entpd1,Casp1,Kcnj3,Gstp1,Adamts7,Ccr2,Ppard,Pon3,Pappa,Syp,Nes,Arg1,Timp1,Pxdn,Pmf1, Mbp,Gja5,Calcrl,Myd88,Ncf1,Serpinf2,Tfpi |
| **sleep disorders** | **86 genes**  F7,Notch1,Lmna,Ednrb,Gata4,Kcnma1,Fbn1,Bdnf,Serpine1,Hp,Nlrp3,Ccl2,Foxp2,Mmp9,Shank3,Gnb3,Fas,Cp,Adipoq,Acta2,Igfbp3,Avp,Cbs,Ass1,Tubb3,Myh9,Lox,Icam1,Tac1,Mapt,Gfap,Myh7,Tshr,F5,Serpinc1,Lepr,Flna,Hbb,Nos3,Tf,Il6,Tlr4,Alb,Sele,Fos,Ace,Tnni3,Ren,Wfs1,Uchl1,Itgb3,Eln,Actb,Flnc,Ednra,Esr1,Gria3,Tspo,Dnah8,Asl,Mpl,Pdgfrb,Nppb,Gdnf,Enpp1,Tnf,Npy,Adrb1,Gcg,Mmp2,Ldlr,Vdr,Dguok,Nkx21,Lipc,Wnk3,Ada,Agt,Atrx,Kcnh2,Tgfbr1, Ache,Vegfa,Hmox1,Il10,Tgfb1 |
| **cognitive disorder&** **sleep disorders** | **23 genes**  Grn,Nefl,Abcc8,Il18,Mpo,Stat3,Igf1,Kng1,Tp53,Chat,Apoe,Vwf,Ttr,Abcc9,Selp,Park7,Edn1,Tgfbr2,Notch3,Apoa1,Rbfox3,Cd36,Ngf |

**Table S6**: Overlapped genes between stroke and Inflammation/Apoptosis related genes

| **IR vs S** | |
| --- | --- |
| **Inflammation** | **64 genes**  Grn,Procr,Adam17,Plat,Kng1,Ccr2,Vwf,Pappa,Ttr,Selp,Arg1,Mbp,Myd88,Ncf1,Apoa1,Ednrb,Fbn1,Serpine1,Calca,Ptx3,Hp,Cp,Sell,Cbs,Plg,Samhd1,Tac1,Gfap,Myh7,Tshr,F5,Serpinc1,Flna,Tf,Ucp2,Alb,Thbd,Sele,Cybb,Ace,Tnni3,Tek,Mmp13,Itgb3,Eln,Socs1,Ccl3,Cyba,Nampt,Ednra,Pf4,Nppb,Cfh,C3,Eng,Proc,Tgfb3,Cxcl1,Ldlr,Mmp3,Agt,Mmp12,Ptpn22,Mmp8 |
| **Apoptosis** | **15 genes**  Park7,Acta2,Casp9,Kitlg,Twist1,Itgb1,Actb,Tspo,Esr2,Pdgfrb,Hdac9,Vhl,Prkch,Bmp7, Ache |
| **Inflammation & Apoptosis** | **74 genes**  Il18,Mpo,Stat3,Igf1,Casp1,Tp53,Gstp1,Ppard,Apoe,Timp1,Edn1,Tgfbr2,Cd36,Ngf,Ctsb,Notch1,Nod2,Lmna,Ntrk1,Bdnf,Fn1,Nlrp3,Sod2,Timp3,Bcl2,Ccl2,Mmp9,Csf3,Itgb2,Fas,Spp1,Flt1,Angpt2,Thbs1,Adipoq,Igfbp3,Olr1,Ccr5,Icam1,Mapt,Ptgs1,Lcn2,Hbb,Nos3,Il6,Tlr4,Fos,Kdr,Lgals3,Ptgs2,Cd14,Ifih1,Esr1,Gdf15,Cd40,Xdh,Elane,Gdnf,Tnf,Cxcr4,Gcg,Mmp2,Adm,Tlr2,Vdr,Csf2,Cav1,Ada,Srebf1,Tgfbr1,Vegfa,Hmox1,Il10,Tgfb1 |
